# Synthesis of platform chemicals from ground tire rubber waste by means of ozonolysis

**DOI:** 10.1039/d6ra05012c

**Published:** 2026-07-20

**Authors:** Yanou Fishel, Sébastien Maricaux, Filip Lemière, Pieter Billen, Christophe M. L. Vande Velde

**Affiliations:** a Intelligence in Processes, Advanced Catalysts and Solvents (iPRACS), University of Antwerp Groenenborgerlaan 171 2020 Antwerp Belgium Yanou.Fishel@uantwerpen.be; b Biomolecular & Analytical Mass Spectrometry (BAMS), University of Antwerp Groenenborgerlaan 171 2020 Antwerp Belgium

## Abstract

Tires are adequately engineered to be mechanically and chemically durable. This makes them difficult to recycle. We have demonstrated the application of ozonolysis as a recycling technique comprising a three-phase (solid–liquid–gas) reactor system with granulated waste tire at −30 °C and atmospheric pressure in order to obtain levulinic and succinic acid. Under these conditions, on average 74 wt% of organic product and 34 wt% of carbon black residue with respect to the original starting mass of crumb rubber was recovered. Both of the identified main product molecules are coined as platform chemicals, implying their broad range of applications. No more polymer matrix was detected in the carbon black residue after the reaction. This presents a radically new potential chemical recycling technique for rubber, allowing the green synthesis of platform chemicals.

## Abstract 2

One tire, two value streams, zero waste. Putting a brake on tire waste by re-routing a recalcitrant thermoset into biocompatible platform acids and intact carbon black filler through sustainable low temperature oxidative deconstruction.

## Graphical abstract

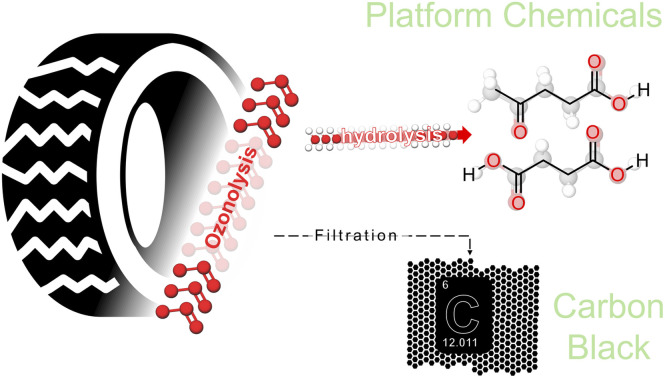

## 1 Introduction

Tires are indispensable facilitators of global transportation and commerce. In 2024, 3.9 million tonnes^1^ reached their end of life in Europe. In Belgium alone, this was equal to 92.5 kilotonnes.^2^ A substantial amount that underscores the critical need for effective recycling methods to mitigate environmental impact and resource depletion.

Depending on the tire type and component, natural rubber or synthetic rubbers (*i.e.*, styrene–butadiene copolymers and butadiene rubber) are incorporated with sulfur in order to vulcanize the elastomers, forming a robust thermoset tire matrix. To enhance vulcanization efficiency, inorganic accelerators such as zinc oxide and organic accelerators such as derivatives of stearic acid, benzothiazole, thiocarbanilide, guanidine or morpholine are added.^3,4^ Fillers such as carbon black and silica are added to increase the rigidity of the thermoset structure, protect against UV degradation, and dissipate heat. Anti-ozonants, including hydrocarbon waxes and *p*-phenylenediamine derivatives,^5^ are compounded to prevent ozone cracking. Additionally, steel wiring and textile fibers such as nylon, polyester, or aramid are incorporated to reinforce the overall structure. Variations in tire formulations exist based on application, brand, and model, each with unique compositions. Generally, the composition of an average passenger car is summarized in Table 1, as derived from literature.

**Table 1:** Average passenger car tire composition derived from ref. 6–11

Passenger car tire	
Rubber elastomer	43 ± 3 wt%
Natural rubber	19 ± 4 wt%
Synthetic rubber	24 ± 2 wt%
Steel	13 ± 3 wt%
Textile	4 ± 1 wt%
Fillers	27 ± 3 wt%
Antioxidants, antiozonants, curing agents	12 ± 2 wt%

Furthermore, the formulations of tires are confidential information to each company to provide a competitive advantage. This adds an additional layer of complexity for recycling as no accurate feedstock input can be easily determined. A rough, approximate overview based on patent literature of the rubber balance in different parts of a tire is given in Table 2 in order to estimate the average rubber composition of rubber granulate.

**Table 2:** Types of rubber per tire part based on ref. 9 and 12–19

Tire part	wt%	NR	SBR	BR	IIR	HIIR^a^
Tread	32.6	29 ± 14	64 ± 17			
Base	1.70	100				
Sidewall	21.9	55 ± 15		51 ± 10		
Inner liner	12.4		36 ± 5			64 ± 5

a Halobutyl rubber. All values in wt%.

Tires are indispensable in a wide range of transportation applications because of their mechanical and chemical resilience, which contribute to their long service life. However, this durability presents significant challenges for recycling efforts in Europe.

Initially, waste tires were either reused or landfilled, constituting the primary waste management strategies. Reuse involved repurposing tires, either in their entirety or in parts, for applications such as tarp weights or components of children's swings, among other creative uses. However, the vast quantity of waste tires severely limits the scalability of these applications.^20^ Alternatively, truck tires can undergo re-treading, where the profile and shoulder of the tire are shaved and replaced by curing a new layer on the tire or pre-curing and gluing it on top, thus extending their lifecycle.^21^

Landfilling, *i.e.*, the collective storage of tires in registered plots or through illegal dumping, presents significant environmental challenges.^22–25^ Tires are difficult to compress, resulting in substantial space requirements and large void volumes between them. These voids can collect rainwater, providing breeding grounds for mosquitoes and bacteria, the latter producing methane gas and occasionally leading to auto-ignited fires.^26–28^ These adverse effects underscore the need for more sustainable waste management solutions.

Since the ban on landfilling tires in Europe in 2003,^29^ tire recycling has gained significant momentum. Initially, tires undergo mechanical recycling, where they are shredded into granulate, known as crumb rubber; ground tire rubber (GTR). During this process, steel wires are removed magnetically, and a substantial portion of textiles is washed away. The resulting crumb rubber finds applications as filler material in artificial turf, elastic flooring (*e.g.*, playgrounds, tennis courts), and is also incorporated into asphalt.^30,31^ However, recent studies have indicated that using crumb rubber in asphalt can lead to the leaching of zinc and organic compounds into the aquatic environment.^22,32–37^ The conversion of tires into rubber granulate is in itself a recycling method, but additionally it is an essential stepping stone toward more intensive valorization processes.

Crumb rubber can be further valorized through (thermo-)chemical recycling techniques such as devulcanization, rubber reclaiming, and pyrolysis.^38^

The primary challenge with these recycling technologies is the material depreciation that often occurs, resulting in a predominantly linear tire lifecycle. This is particularly evidenced by their improvised use in applications as a means to extend their lifecycle (*e.g.*, as a tarp weight or their use in playgrounds). Devulcanization and reclaiming techniques typically yield low outputs and produce semi-finished products that must be compounded with virgin materials, ultimately performing less effectively than pure virgin materials. Pyrolysis of tires generates a wide array of compounds, many of which are difficult to recover (*e.g.*, hydrogen gas, methane, ethane) due to the nature of the process. While the carbon black obtained from pyrolysis is of potentially high value as a recycled substitute^39–43^ for virgin carbon black, it necessitates an additional purification step to remove non-volatile fillers such as silica, zinc oxide, and other metal oxides. This is a clear signal that multiple approaches will be required to tackle the recalcitrant waste stream to optimize its reapplicability.

**Fig. 1:**
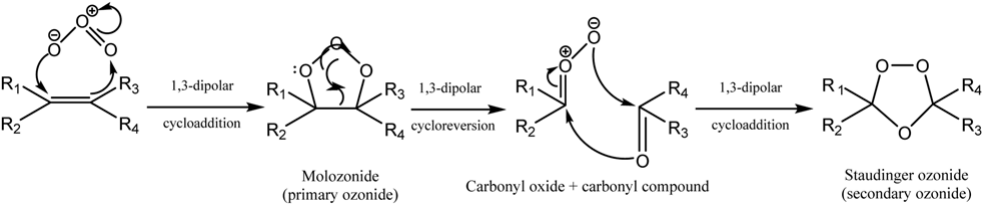
Criegee mechanism of ozonolysis.^45^

In this work, we explore ozonolysis as a recycling technique. Atmospheric ozone attacks tires in ambient conditions, which is why antiozonants have been added to the formulation since the 1950's to prevent them from cracking. Ozonolysis, discovered by Harries^44^ in 1905 and further elucidated by Criegee^45,46^ and Bailey,^47^ involves the selective oxidation of compounds at positions of unsaturation. The mechanism, as described by Rudolph Criegee^45^ and depicted in Fig. 1, consists of three consecutive steps (1,3-dipolar cycloaddition, 1,3-dipolar cycloreversion, and 1,3-dipolar cycloaddition), leading to the formation of molozonide, carbonyl oxide, and finally Staudinger ozonide reactive species. We decided to build on this known phenomenon and deploy it further to attempt to completely fragment the rubber. It is important to note that Staudinger ozonide species (secondary ozonide) are unstable and must be handled with care,^48,49^ as they can thermally decompose violently, resulting in explosions.^50^ In addition, ozone gas is a toxic gas that can cause irritation and damage to mucous and respiratory tissue at low concentrations (>0.1 ppm). Finally, the combined use of flammable organic solvents and oxidizing gas streams (oxygen or ozone gas) poses an inherent fire and explosion risk. The vapor pressure of the solvent should be considered when designing reaction conditions, and all potential ignition sources must be removed.

Secondary ozonides can be further processed into carboxylic acids, aldehydes, ketones, or alcohols, depending on whether oxidative or reductive conditions are applied. In laboratory settings, ozone for these reactions is typically generated *in situ*, using an ozone generator, which produces ozone by applying a high voltage discharge to oxygen gas, resulting in singlet oxygen that subsequently forms ozone. The ozone is supplied as a 1–10 weight percent (w%) mixture in an oxygen stream. Excess ozone and oxygen exit the reaction vessel, where ozone either decomposes spontaneously or is passed through a decomposition catalyst such as manganese dioxide (MnO_2_).^51–54^

The ozonolysis of ground tire rubber necessitates a three-phase (solid–liquid–gas) system, as vulcanized rubber is insoluble in organic solvents. Mass transfer is a significant challenge in such systems. Additionally, as the polymer fragments, the solution turns black due to the release of carbon black, eliminating visual cues typically used to monitor the reaction, such as the solution turning blue when saturated with ozone or the use of Sudan III to indicate reaction completion in traditional olefin ozonolysis.^49,55^ The formation of polymeric ozonides^56^ or flocculation, as seen in polybutadienes,^57^ is also obscured. Ground tire rubber presents a complex substrate compared to typical olefin ozonolysis reactions, which usually involve single compounds with few olefinic bonds. Theoretically, cleaving rubber at sequential olefin positions yields bifunctional molecules with oxygenated moieties at both ends, necessitating a compatible work-up method. Traditional work-ups involving reagent addition followed by liquid–liquid extraction, where products are obtained in the organic phase and waste by-products in the aqueous phase, are unsuitable if the products are highly water-soluble.

This paper introduces a novel strategy to address these challenges, demonstrating a potential breakthrough in rubber depolymerization platform technology. Furthermore, the ozone production in ozonolysis is entirely electrically driven, allowing transitioning to renewable energy sources for this technology.

### 1.1 Ozonolysis of natural rubber

In 1905, Harries^44,46^ pioneered the ozonolysis of rubber, laying the groundwork for the structural elucidation of rubber and introducing ozone into the organic chemistry toolkit, hence the term “Harries ozonolysis”. Harries achieved a 26% yield of levulinic acid. Subsequent studies by Chakravarty in 1967 (ref. 58) and McSweeney in 1968 (ref. 59) explored the ozonolysis of both unvulcanized and vulcanized natural rubber in chloroform and chloroform/ethanol solvents, respectively. From unvulcanized natural rubber Chakravarty^58^ reported a 23% yield of levulinic acid, while McSweeney^59^ documented a 75–85% yield of levulinic acid 2,4-dinitrophenylhydrazone indicating that the ozonolysis is stoichiometric but some of the formed levulinic acid is further oxidized. Additionally, Chakravarty noted the formation of sulfuric acid from vulcanized samples, as well as formic acid from poly(3,4-)isoprene and acetic acid from the decomposition of levulinic aldehyde.^58^

In 2000, Nor *et al.*^60^ and later in 2021, Banan *et al.*^61^ have both investigated the use of ozonolysis to produce high molecular weight (MW) oligomers from unvulcanized natural rubber. Nor *et al.*^60^ examined the changes in MW and functionality during the ozonolysis reaction using Fourier transform infrared spectroscopy (FTIR) and gel permeation chromatography (GPC). Their findings indicate that the most significant reduction in MW (from 27 000 to 5000 g mol^−1^) occurs within the initial minutes of ozonolysis, with oligomers of less than 900 g mol^−1^ obtained after 20 minutes. Banan^61^ successfully obtained liquid natural rubber (LNR) with a MW of 2368 g mol^−1^ and hydroxyl-terminal groups under optimized conditions, proposing its use as a polyol source for polyurethanes. However, attempts to achieve similar results with natural rubber in the latex phase were unsuccessful.

### 1.2 Ozonolysis of synthetic rubber

In Bailey's^62,63^ monographic series of ozonation in organic chemistry he touches on the relative reaction rates of olefins *versus* aromatic (and other moieties). He reports a higher reaction rate for olefinic-than aromatic systems, as was the basis for an early synthesis of vanillin from isoeugenol. Furthermore, it is summarized that ozonolysis of substituted benzene-rings often leads to scission at the ipso-carbon (*e.g.*, ozonolysis of β-phenylpropionic acid, phenylacetic acid, and benzoic acid afforded succinic acid, malonic acid, and oxalic acid, respectively, along with formic acid and glyoxal). Finally, the reaction rate increases with greater activation of the aromatic ring due to electron-donating substituents.

Couve *et al.*^64^ demonstrated in 1997 that star-branched styrene–butadiene copolymers dissolved in dichloromethane can be cleaved at butadiene unsaturations through ozonolysis, although only a modest decrease in molecular weight was observed (−52.45% over 5 h). Alekseeva and Belitzkaya,^65^ followed by Rabjohn *et al.*,^66^ investigated the ozonolysis of styrene–butadiene copolymers for structural determination. They primarily obtained succinic acid, along with formic acid and the methyl esters of succinic acid, phenyladipic acid, and diphenylsuberic acid from ozonolysis in chloroform at 0 °C. However, the mass balance in their study showed significant losses, and the sole characterization technique used was elemental analysis, which is prone to inaccuracies.^67^ Formic acid was determined by titration, which can yield positive errors if compounds like formaldehyde or succinaldehyde are present.

Tanaka *et al.*^68^ studied the sequence length distribution of styrene-butadiene copolymer using ozonolysis and GPC. They initially applied their method to polybutadiene (*cis*-1,4: 96%; *trans*-1,4: 2%; 1,2: 2%) in dichloromethane at −40 °C, decomposing the ozonide with zinc and acetic acid to aldehydes, and later with lithium aluminium hydride to alcohols. They reported the formation of succinaldehyde (or 1,4-butanediol) from 1,4–1,4 sequences and 3-formyl-1,6-hexanal from 1,4–1,2–1,4 sequences in smaller quantities. Complete ozonolysis was indicated by the absence of olefinic proton signals in proton nuclear magnetic resonance spectroscopy (^1^H-NMR). When the solvent was changed from dichloromethane to chloroform, carbon tetrachloride, diethyl ether, *n*-hexane, and carbon disulfide, poor ozonolysis yields and higher molecular weight products were obtained, besides succinaldehyde. Attempts to apply the same method to styrene oligomers and polystyrene showed no change in molecular weight, indicating that styrene units in SBR are inactive to ozone under these conditions. When the method, followed by ozonide work-up with lithium aluminium hydride, was applied to styrene–butadiene copolymer, they primarily obtained 3-phenyl-1,6-hexanediol (from 1,4-St-1,4 sequences) and compounds from linkages containing the 1,2-sequence. They found that the copolymer contained relatively few styrene-1,2-linkages and rather long styrene sequences (up to 35 styrene units), with some longer oligomers almost identical to polystyrene in ^1^H-NMR.

In 2010, Yang *et al.*^57^ studied the ozonolysis of polybutadienes in chloroform and chloroform/ethanol mixtures. Ozonolysis was carried out at 30 °C on mixtures of elastomer and ethanol in chloroform (1 g elastomer/100 ml CHCl_3_), with elastomer-to-ethanol weight ratios of 1 : 1, 1 : 2, and 1 : 3 (w/w). They reported that ozonolysis efficiency was higher when 1 w/v% ethanol was added but decreased again with higher ethanol concentrations (≥2 w/v%). They observed that in the first 20 min of ozonolysis, the decrease in MW followed the same trend with or without ethanol, but after 30 min, side reactions such as cross-linking began to occur when no ethanol was present. Additionally, increased concentrations of ethanol did not affect the final product functionality, as determined by GPC, FTIR, and ^1^H-NMR.

### 1.3 Platform chemicals

In 2004, Werpy and Peterson^69^ published a list of 12 biologically-derivable platform chemicals: glycerol, 3-hydroxypropionic acid, C4 acids (succinic acid, fumaric acid, malic acid), 3-hydroxy-butyrolactone, aspartic acid, itaconic acid, xylitol, levulinic acid, glutamic acid, sorbitol, glucaric acid and 2,5-furandicarboxylic acid. In 2010,^70^ the top 10 of these chemicals were revisited and changed (to ethanol, furans, glycerol and derivatives, biohydrocarbons, lactic acid, succinic acid, hydroxypropionic acid (and aldehyde), levulinic acid, sorbitol, xylitol). These chemicals are to replace the current fossil-based platform chemicals: ethylene, propylene and benzene. The idea of platform chemicals is that a small number of chemical intermediates can be each converted to a large number of chemical products.^71^ On this list are levulinic acid and succinic acid. Levulinic acid can be produced from hydroxymethylfurfural (HMF), which is formed through the dehydration of hexose sugars. It can be transformed into substituted pyrrolidones, lactones, levulinate esters and other compounds.^70^ Succinic acid is obtained from the fermentation of sugars using *Anaerobiospirillum succiniciproducens* or *Anaerobiospirillum succiniciproducens*^72,73^ amongst others. Succinate esters are precursors to existing petrochemical products such as 1,4-butanediol, tetrahydrofuran, γ – butyrolactone and other pyrrolidinone derivatives.^70^ These two chemicals were also obtained from ozonolysis of rubbers in past research. This makes waste rubber a possible alternative source to biomass.

## 2 Methods

Detailed information on materials and analytical methods is provided in the SI Sections I and II.

### 2.1 Experimental setup

The ozonolysis setup consisted of a magnetic stirrer-equipped double-walled reaction vessel. Through a Pyrex fritted gas dispersion tube (Sigma-Aldrich, porosity 3) ozone was introduced. The outlet of the adapter was connected to a silicone hose, which exhausted into the back of the fume hood. The setup was cooled using an external circulator.

### 2.2 Ozonolysis of ground tire rubber

Approximately 1 g of rubber was suspended in 160 ml of chloroform (or dichloromethane or a mixture of 10 v% methanol/dichloromethanem) and allowed to swell overnight before the reaction. An oxygen stream containing approximately 3% ozone by mass was bubbled through the heterogeneous mixture at a rate of approximately 0.94 L min^−1^ (equivalent to 0.8 mmol O_3_/min) at −30 °C for an average duration of 20 min per gram of rubber. After the reaction, residual ozone was purged from the mixture with oxygen for 5–10 min. The contents of the reactor were then transferred to an Erlenmeyer flask and allowed to warm to room temperature.

After the rubber granulate was allowed to swell in the solvent overnight, the solvent acquired a brown color due to leached components. This color disappeared immediately upon starting the ozonolysis. Concurrently, the reaction medium turned black as carbon black was released from the polymer matrix. Upon standing, the carbon black settled and any remaining rubber granulate floated on top.

### 2.3 Ozonoylsis of styrene butadiene rubber

Synthos Sprintan 3402 rubber was purified by dissolving into chloroform and adding ethanol until the point of precipitation. The solution containing the any treated distillate aromatic extract (TDAE) was decanted, and the rubber was left to dry to air. Approximately 3 g of purified Synthos Sprintan 3402 was dissolved in 160 ml CHCl_3_ overnight. An oxygen stream containing approximately 3% ozone by mass was bubbled through the heterogeneous mixture at a rate of approximately 0.94 L min^−1^ (equivalent to 0.8 mmol O_3_/min) at RT for an average duration of 1 h. After the reaction, residual ozone was purged from the mixture with oxygen for 5–10 min. The contents of the reactor were then transferred to an Erlenmeyer flask.

### 2.4 Hydrolytic conversion to carboxylic acids

**Fig. 2:**
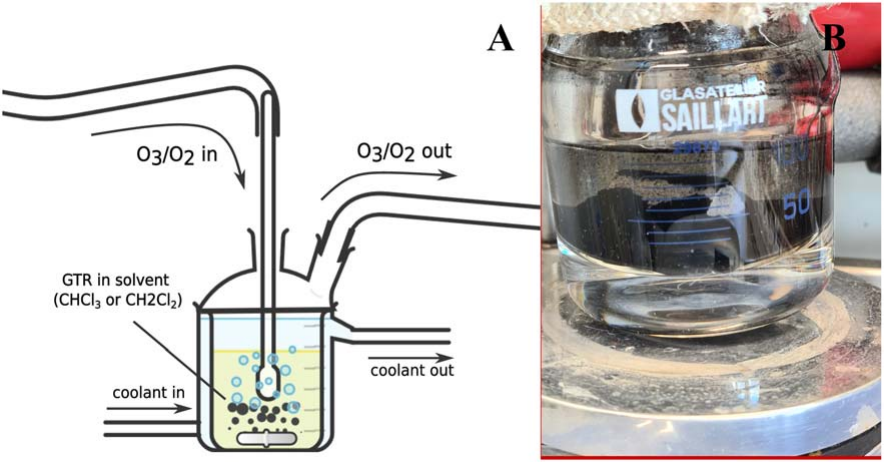
(A): graphical representation of the ozonolysis setup; (B): post-ozonolysis mixture with carbon black settled at the bottom of the reactor vessel.

The post-ozonolysis reaction mixture was allowed to stand, resulting in residual rubber floating and released carbon black settling at the bottom (see Fig. 2B). The mixture was filtered using a Thermo Fisher 0.45 micron PTFE-membrane filter with a membrane filtration apparatus. The residue was washed multiple times with methanol until clear and filtered over a 0.45 micron nylon membrane filter. The chloroform (and in some cases, part methanol) was distilled off, and distilled water was gradually added. Once the original volume of reaction solvent had been replaced by water, the setup was reconfigured for reflux, and heating continued under reflux conditions. The absence of ozonides or peroxides was confirmed when iodine was no longer released after a few drops of the refluxing mixture were mixed with a potassium iodide solution. Reflux was then ceased, and further concentration *in vacuo* resulted in a heterogeneous crude mixture, consisting of a brown viscous syrup with some crystalline precipitation.

### 2.5 Conversion to carboxylic acids and aldehydes through base treatment

The post-ozonolysis procedure mirrors the hydrolytic work-up. Following filtration, triethylamine (99%) was added dropwise to the post-ozonolysis reaction mixture and allowed to stir. The absence of iodine release upon mixing a reaction aliquot with potassium iodide indicated the completion of the reaction. Excess triethylamine was removed on a rotary evaporator and the mixture was further concentrated. A heterogeneous crude mixture comprising a brown viscous syrup with some small crystals, including triethylamine *N*-oxide crystals was obtained.

## 3 Results

### 3.1 Ground tire rubber product analysis

After allowing the rubber granulate to swell in the solvent overnight, the solvent acquired a brown color due to leached components. This color disappeared immediately upon starting the ozonolysis, and these compounds were no longer detected by gas chromatography-mass spectrometry (GC-MS), indicating their degradation within the first minutes (see SI Fig. S6 & S7).

Initially, the ozonolysis of ground tire rubber was conducted multiple times on 1 g and 5 g scales in dichloromethane and dichloromethane with 10 v% ethanol. On average, 59 ± 4 wt% product and 51 ± 7 wt% residue were obtained in dichloromethane. The reaction was also tested with methanol but due to its slightly better swelling characteristics for GTR, ethanol was used as co-solvent. In dichloromethane with 10 v% ethanol, 76 ± 7 wt% product and 45 ± 8 wt% residue were obtained (see SI Table S1 for full data). The increase in mass (exceeding 100 wt%) is attributed to the incorporation of a significant amount of oxygen into the reaction products. The theoretical mass obtained at 100% conversion of the rubber components of the average passenger car tire (based on Table 1, excluding any steel or textile since those are removed during granulation and assuming all synthetic rubber is butadiene) would equal 150 wt% of the starting mass. This does not account for over-oxidation to CO_2_ or oxidation of any filler materials. An actual mass balance is challenging due to the unknown exact composition of the waste tires and their precise amount of olefinic bonds.

**Fig. 3:**
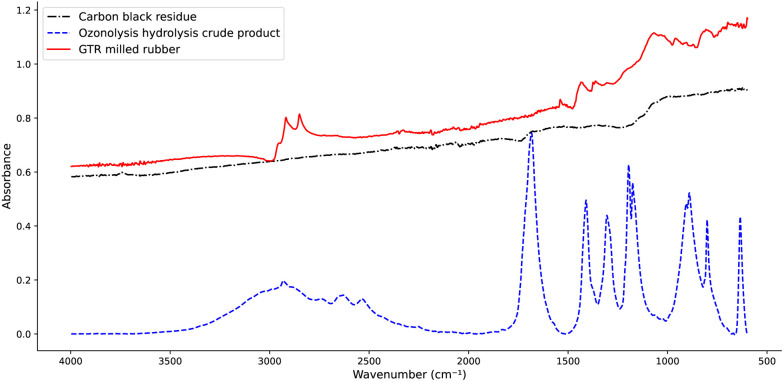
FTIR spectra of ground tire rubber (red), residue (black, dot/dashed) and crude product (blue, dashed) obtained from ozonolysis–hydrolysis reaction process.

**Fig. 4:**
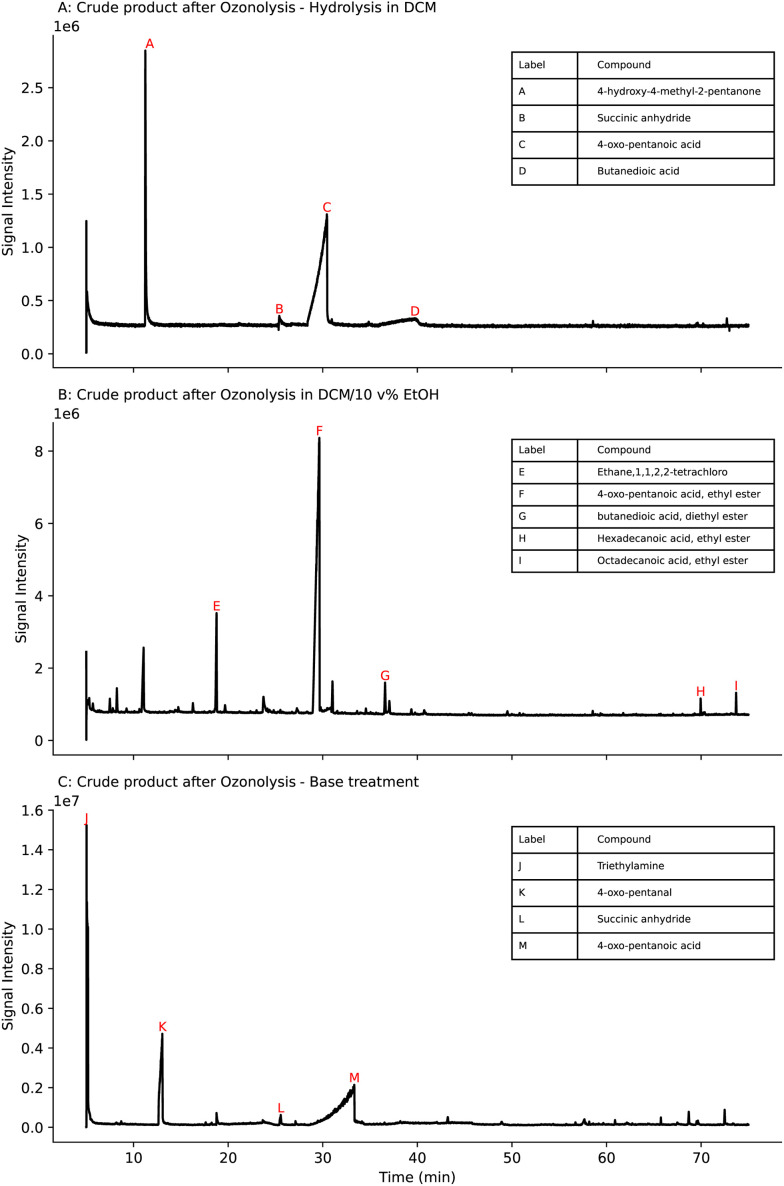
GC-MS chromatogram of crude product obtained from ozonolysis–hydrolysis (A) reaction in dichloromethane, crude product after ozonolysis in dichloromethane with 10 v% ethanol (B) and of crude reaction product obtained from ozonolysis-base treatment (C) reaction process in dichloromethane.

When an alcohol was used as co-solvent, the mass increase was further amplified due to the formation of alkyl esters instead of carboxylic acids, *e.g.*, the formation of methyl esters observed when methanol was used (see Fig. 3B, SI Fig. S9 and Table S1) as seen in Fig. 4B. When the reaction was worked up through base treatment (*i.e.*, triethylamine), the crude product mass was significantly higher (195% compared to the mass obtained from hydrolytic work-ups) due to the presence of remaining triethylamine *N*-oxide that could not be removed on a rotavapor. The crude was characterized directly, without any further purification, as this would be difficult and require multiple steps.

It was later noticed that a lot of the organic product remains adsorbed on the carbon black, thus a new reaction set (*n* = 9) was performed in chloroform and the carbon black residue was washed multiple times with methanol until no more product was detected on GC-MS. On average, 74 ± 5 wt% product and 34 ± 2 wt% residue were obtained. The obtained mass balance distribution remains in accordance with the distribution obtained of reactions in dichloromethane when assuming that a portion of the product was adsorbed onto the carbon black residue. During hydrolysis, the mixture was observed to turn brown, believed to be polymerization through aldol condensation and Michael addition of intermediary polycarbonyls (*e.g.*, 4-oxopentanal prior to further oxidation to levulinic acid) that are formed during the hydrolysis of rubber ozonides.

Fig. 3 presents the FTIR spectra of the rubber material before ozonolysis, the recovered residue, and the crude product mixture obtained after work-up. A clear distinction is evident between the starting material (red) and the reaction products (blue). The starting material exhibits a rising baseline, characteristic of carbon black absorbance across the IR wavelength spectrum. Notable peaks at 2921 cm^−1^ and 2858 cm^−1^ correspond to long paraffinic oils within the rubber structure, which migrate to the surface over time^74^ due to the compression and relaxation of tires during use, acting as a physical barrier against ozone.

The crude ozonolysis product displays a strong asymmetrical peak at 1682 cm^−1^, indicative of various carbonyl groups (ketones and carboxylic acids). This is further supported by peaks at 1409 cm^−1^ (C–H bend adjacent to carbonyl; methylene ketone), 1305 cm^−1^ (carboxylate C–O stretch), and a broad peak between 2800 cm^−1^ and 3400 cm^−1^ (O–H stretch in carboxylic acids). The recovered residue (black) shows a shifted baseline similar to that of carbon black, with no distinguishable peaks, due to its absorbance throughout the IR spectrum.

The GC-MS chromatograms of the products obtained through different work-up methods and active/inert solvent mixtures are shown in Fig. 4A–C. Both work-up methods (hydrolysis and base-treatment) primarily yielded succinic acid (or its anhydride, due to the high injector temperature) and levulinic acid. It is possible that thermally labile compounds decomposed before analysis due to the injector temperature of 320 °C. The work-up with triethylamine also produced the aldehyde of levulinic acid.^75,76^ The change in retention time of levulinic acid is attributed to interactions with triethylamine, causing peak broadening. Succinic acid exhibits a lower response in positive-ion MS compared to levulinic acid, due to poor ionization. Levulinic acid (4-oxopentanoic acid) ionizes more readily, forming methyl and acylium cations, which complicates quantification due to the difference in response. When ethanol was used as a co-solvent, ethyl esters of levulinic acid and mono- and diethyl esters of succinic acid were identified.

Quantification through gas chromatography-flame ionization detection (GC-FID) after esterification of the acids resulted in an average of 46 ± 3 wt% levulinic acid, 4 ± 1 wt% succinic acid was determined over 5 different reaction samples. Palmitic acid and stearic acid were also esterified but were found to be below the limit of quantification of 50 ppm (or equal to a response ratio (analyte/IS) of 0.05) under the dilution and injection conditions used (see SI Fig. S25).

69 wt ± 4.6 wt% was observed to be volatile under 240 °C (SI Fig. S5), as shown through thermogravimetric analysis (TGA) performed on 3 underivatized reaction samples to estimate the aldol-condensed wt% that was inaccessible to derivatization and GC quantification.

Furthermore, energy dispersive X-ray fluorescence (see SI Fig. S26) revealed 1.2 wt% of Zn in the obtained crude product, presumably present as zinc succinate (equal to approx. 3 wt% of the organic product).

### 3.2 Residue content

No polymer matrix was observed through TGA conducted on the obtained residue fraction (see SI Fig. S4), as it would have been pyrolyzed under a nitrogen atmosphere during the pyrolysis step. Subsequently, a combustion step was performed under an air atmosphere, during which 82 wt% of the residue was volatilized. This measurement was repeated using activated charcoal (Norit CN1) as a reference (see SI Fig. S4).

Inductively coupled plasma optical emission spectrometry (ICP-OES) analysis of the mineral matrix determined the composition of 1.4 wt% Zn, 32 wt% Si, and 1.7 wt% S. Additionally, 1.3 wt% Al, 2.0 wt% Ca, 1.7 wt% Cu, and 5.3 wt% Fe were detected (see SI Table S3 for the complete data). The composition is in line with an expected passenger car tire ash, except that the Zn value is lower than expected. This can be clarified by the fact that it is partly present in the organic fraction.

### 3.3 Styrene butadiene rubber analysis

Synthos Sprintan 3402 (15 wt% styrene)^77^ was used to determine the fate of styrene units during ozonolysis. As seen in Table 2 most styrene butadiene rubber is found in the tread of a tire, which is diminished during its life cycle. An aliquot of 2 ml was taken from the reaction mixture prior to and after reaction completion and left to dry as the formed product no longer dissolves in chloroform and would hamper ^1^H-NMR analysis. A viscous clear liquid was obtained after the post-reaction aliquot was left to dry. The reagent Sprintan rubber (0.0192 g) was dissolved in deuterated dichloromethane with 1-docosene as internal standard. It was observed that 13 wt% of styrene was present (see SI Fig. S2 & Table S2). The dried aliquot of the post-ozonolysis sprintan rubber (0.0485 g) was dissolved in a mixture of NaOD/D_2_O with disodium itaconate as internal standard. 7.5 wt% of styrene was observed after ozonolysis (see SI Fig. S3 & Table S2). An approximate 2.5-fold mass increase per 2 ml aliquot was observed while the relative styrene concentration decreased by 42% due to the formation of succinic acid or succinic aldehyde from poly-(1,4-butadiene). Theoretically, the mass is expected to double if solely butadiene-units would be oxidized to succinic acid (Per 100 g Sprintan 3402 consists of 15 g styrene and 85 g butadiene, where the latter would increase to 185 g if oxidized to succinic acid. Additionally, a 1.5-fold increase is expected if solely succinic aldehyde would be formed and a 1.76-fold increase if only secondary ozonides would be formed). The excess mass increase is ascribed to solvent evaporation as the styrene mass in the aliquot increased by approximately 1.44 times (0.0025 g prior to ozonolysis *versus* 0.0036 g post-ozonolysis) where it would be expected to remain constant or decrease if styrene were itself cleaved. When taking both the aliquot mass increase and the styrene mass per aliquot into account, the two do not increase equally. When attributing the increase in aliquot concentration solely to solvent evaporation equal to the increase in styrene mass per aliquot, then the corrected mass increase is 1.76. This falls between the bounds for complete conversion to succinic acid (2.00) and to succinic aldehyde (1.50) as it is unlikely that ozonides remain present. This indicates that styrene units are unaffected by ozonolysis conditions, which is in accordance to the observations of Alekseeva *et al.*^65^ Accordingly, as described in Bailey's monograph,^63^ the aromatic ring is much less susceptible than olefin bonds to partake in the ozonolysis. This observation is corroborated by FT-IR analysis of the obtained product compared to the reagent material (see SI Fig. S1) where aromatic C–H out of plane bending is observed at 700 and 750 cm^−1^ as well as aromatic C=C stretching at 1650 cm^−1^ both prior and after the reaction. Additionally, the obtained reaction product shows a strong asymmetric peak at 1707 cm^−1^, indicative of various carbonyl groups (aldehydes and carboxylic acids) as well as a broad signal at 3000–3600 cm^−1^ in accordance to O–H stretch vibrations of carboxylic acids. A shoulder typical of aldehydes at 2720 cm^−1^ is present but not very pronounced. Additionally, a signal is seen at 9.4 ppm on ^1^H-NMR, confirming the presence of aldehydes, but cannot be fully quantified as they undergo aldol-condensation in the NaOD/D_2_O mixture. Finally, the decrease in *cis*- and *trans*-1,4-butadiene double bonds cannot be established by FTIR analysis as the signals (at 725 and 965 cm^−1^ respectively) are obscured by other signals. However, NMR analysis revealed that the signal at 5.4 ppm, attributed to the internal olefinic protons of 1,4-polybutadiene units (*cis* & *trans*) is absent following ozonolysis, confirming cleavage of the olefinic bonds.

## 4 Discussion

We show in this study that ozonolysis has the potential to become a sustainable method to transform granulated tire waste into platform chemicals. Our findings indicate that ozonolysis, coupled with hydrolysis as a work-up, can effectively disassemble crumb rubber particles into levulinic and succinic acids, along with a solid residue primarily consisting of carbon black, silica, and zinc. Levulinic and succinic acids have broad application potential as platform chemicals for polymers (*e.g.*, poly(4-hydroxy-valerate) and polybutylene succinate (PBS)), solvents (*e.g.*, tetrahydrofuran), and as feedstock for 1,4-butanediol production. The anti-ozonants decompose within the first minutes of ozonolysis due to the large excess of ozone, posing minimal obstruction to the method (see SI Fig. S6–S8 & S14–S16).

However, the polymerization that takes place during hydrolysis remains an obstacle as it leads to a considerable amount of product loss. Possible remedies lie in the development of adequate alternative work-up methods. Additionally, the quantification of succinic acid showed a high standard deviation. Within the product mixture, succinic acid forms a solid phase that is heterogeneous with respect to the remaining viscous liquid product, making representative sampling inherently difficult and introducing substantial variability.

Finally, side reactions from the solvent were detected. When dichloromethane was used, ozone abstracted hydrogen in small amounts, resulting in dichloromethyl and hydroxymethyl radicals (the latter in the case when methanol was used as a co-solvent). These radicals propagated and terminated to form side products such as 1,1,2-trichloroethane (see SI Fig. S8, S9 & S18), 1,1,2,2-tetrachloroethane (see SI Fig. S8, S9 & S19), and 2,2-dichloroethanol (see SI Fig. S9 & S22). In the pursuit of green chemistry, minimizing side reactions, especially with solvents, or finding alternative gains (*e.g.*, providing an extra small product stream) is essential.

## 5 Conclusions

This study demonstrates the feasibility of ozonolysis as a route to chemically valorize ground tire rubber waste on a laboratory scale. Using a three-phase (solid–liquid–gas) system applied to real granulated tire waste on 1 g and 5 g scales, an average of 74 wt% product and 34 wt% carbon black residue were recovered relative to the initial crumb rubber mass when the reaction was performed in chloroform. The product fraction comprised 46 wt% levulinic acid and 4 wt% succinic acid (34 and 3 wt% per gram of crumb rubber, respectively) as determined by GC-FID. These are both recognized platform chemicals with broad downstream application potential. The reaction was selective to olefins in that no clear cleavage of aromatic moieties was observed through experiments on pure styrene-butadiene rubber in which styrene units remained unaffected. The concentration of aromatic synthetic rubber in ground tire rubber is too small to observe this explicitly. Although tires are specifically formulated with antiozonants to resist ozone attack, these were found to decompose within the first minutes of reaction under the applied ozone excess, posing no meaningful obstruction to depolymerization. The carbon black residue retained no detectable polymer matrix and consisted of 82 wt% inorganic carbon with an 18 wt% mineral fraction, including 1.4 wt% zinc, 32 wt% silicon, and 5.3 wt% iron. Hydrolysis proved a compatible and sustainable work-up for the resulting ozonides. Two limitations were identified that constrain the achievable yield: polymerization of intermediary polycarbonyls during hydrolysis, which accounts for appreciable product loss, and solvent-derived side reactions, which are counteractive to the green chemistry aims of the process.

## Author contributions

Yanou Fishel: writing – review and editing, writing – original draft, visualization, validation, methodology, investigation, formal analysis, data curation, conceptualization, funding acquisition. Sébastien Maricaux: writing – review & writing – original draft, visualization, methodology, investigation, formal analysis, conceptualization Filip Lemière: methodology, conceptualization, validation Pieter Billen: writing – review and editing, writing – original draft, visualization, validation, supervision, resources, project administration, methodology, investigation, funding acquisition, formal analysis, data curation, conceptualization.Christophe M. L. Vande Velde: writing – review and editing, writing – original draft, visualization, validation, supervision, resources, project administration, methodology, investigation, funding acquisition, formal analysis, data curation, conceptualization.

## Conflicts of interest

There are no conflicts to declare.

## Supplementary Material

RA-OLF-D6RA05012C-s001

## Data Availability

The data supporting this article have been included as part of the supplementary information (SI). Supplementary information is available. See DOI: https://doi.org/10.1039/d6ra05012c.
