# Supplementary material for: Synthesis of platform chemicals from ground tire rubber waste by means of ozonolysis
Source: RSC Adv. 2026 Jul 20;16(38):41387–95. doi: 10.1039/d6ra05012c (PMC13382223; doi:10.1039/d6ra05012c)
Supplement: Supplementary file 1 [file RA-016-D6RA05012C-s001.pdf]

Supplementary Information (SI) for RSC Advances.
This journal is © The Royal Society of Chemistry 2026

Supporting information for

SYNTHESIS OF PLATFORM CHEMICALS FROM GROUND TIRE RUBBER WASTE

Yanou Fishel[a,], Sébastien Maricaux[a], Filip Lemière[b], Pieter Billen[a], Christophe Vande Velde[a]*

[a]iPRACS, Faculty of Applied Engineering, University of Antwerp, Groenenborgerlaan 171, Antwerpen 2020, Belgium.

[b]BAMS, Faculty of Science, University of Antwerp, Groenenborgerlaan 171, Antwerpen 2020, Belgium.

*corresponding author email: Yanou.Fishel@uantwerpen.be

Contents

MATERIALS 2
ANALYTICAL METHODS 3
EXPERIMENTAL RESULTS 5
ANALYSIS RESULTS 6
FOURIER TRANSFORM INFRARED SPECTROSCOPY 6
^{1}H-NUCLEAR MAGNETIC RESONANCE SPECTROSCOPY 6
THERMOGRAVIMETRIC ANALYSIS 8
INDUCTIVE COUPLED PLASMA – OPTICAL EMISSION SPECTROMETRY ANALYSIS 9
GAS CHROMATOGRAPHY – MASS SPECTROMETRY ANALYSIS 10
GAS CHROMATOGRAMS 10
MASS SPECTROMETRY COMPOUND TABLES 13
GAS CHROMATOGRAPHY – FLAME IONIZATION DETECTION 20
ENERGY DISPERSIVE X-RAY FLUORESCENCE 21

Table S1: Experimental data of ozonolysis in DCM (1-7), DCM/10v% ethanol (8-16) and $CHCl_3$ (17-24) 5
Table S2: ^{1}H-NMR quantification data of Sprintan 3402 SBR prior to ozonolysis in $CDCl_2$ (internal standard = 1-docosene) and post ozonolysis in NaOD/D_2O (internal standard = sodium itaconate) 7
Table S3: ICP-OES result of mineral fraction of ozonolysis carbon black 9

Figure S1: FTIR spectra of Sprintan 3402 SBR before (red, solid), and after (blue, dashed) ozonolysis 6
Figure S2: ^{1}H-NMR spectrum of Sprintan 3402 SBR prior to ozonolysis in $CDCl_2$ with 1-docosane added as internal standard 6

Figure S3: ^{1}H-NMR spectrum of Sprintan 3402 SBR following ozonolysis in NaOD/D_2O with Sodium Itaconate added as internal standard 7
Figure S4: TGA plot of activated charcoal (Norit CN1) (blue), obtained carbon black (orange) and ground tire rubber (green) as well as the remaining wt% at 600 °C after pyrolysis under N_2 atmosphere and at 800 °C after combustion under air 8
Figure S5: TGA plot of heating loss of average of 3 ozonolysis-hydrolysis products heated to 240 °C under 60 mL/min N_2 atmosphere 8
Figure S6: GC-MS result of leachates from crumb rubber 10
Figure S7: GC-MS result of ozonolysis reaction in DCM after swelling overnight, before reaction start 11
Figure S8: GC-MS result of ozonolysis reaction in DCM after swelling overnight, after 15 min of reaction 12
Figure S9: GC-MS result of ozonolysis reaction of crumb rubber in DCM/10v% methanol . 13
Figure S10: Mass spectrum of triethylamine and library reference spectrum 13
Figure S11: Mass spectrum of succinic anhydride and library reference spectrum 14
Figure S12: Mass spectrum of succinic acid and library reference spectrum 14
Figure S13: Mass spectrum of levulinic acid and library reference spectrum 15
Figure S14: Mass spectrum of benzothiazole antiozonant and library reference spectrum 15
Figure S15: Mass spectrum of N1-(4-methylpentan-2-yl)-N4-phenylbenzene-1,4-diamine - antiozonant and library reference spectrum 16
Figure S16: Mass spectrum of 4,4'-((p-phenylene)diisopropylidene)diphenol - antiozonant and library reference spectrum 16
Figure S17: Mass spectrum of diethyl phthalate - plasticizer and library reference spectrum 17
Figure S18: Mass spectrum of 1,1,2-trichloro-ethane - DCM side product and library reference spectrum 17
Figure S19: Mass spectrum of 1,1,2,2-tetrachloro-ethane - DCM side product and library reference spectrum 17
Figure S20: Mass spectrum of n-hexadecanoic acid - oxidized physical anti-ozonant wax and library reference spectrum 18
Figure S21: Mass spectrum of 2,2-dichloroethanol - DCM and methanol side product and library reference spectrum 18
Figure S22: Mass spectrum of 2,5-dimethoxy-tetrahydrofuran and library reference spectrum 18
Figure S23: Mass spectrum of methyl levulinate and library reference spectrum 19
Figure S24: Mass spectrum of dimethyl succinate and library reference spectrum 19
Figure S25: GC-FID 50-2000 ppm calibration curve of Methyl levulinate (blue, triangle); Monomethyl succinate (orange, dash), Dimethyl succinate (green, square), Methyl palmitate (light blue, diamond), Methyl stearate (purple, circle) relative concentration to 10000 ppm IS (methyl decanoate) to relative area 20
Figure S26: ED-XRF results of crude ozonolysis product indicating the presence of 1.2 wt% Zn 21

MATERIALS

Ozone was produced with a Purusaqua O3-5000B koi pond ozone generator. Oxygen (99.995%) from Air Liquide is passed through the generator at a flow rate of 0.94 L/min and

pressure just above atmospheric pressure, generating an ozone flow of 0.8 mmol/min, measured with a UV-ozone analyzer (Anseros Ozomat GM 6000 OEM in the 200 g O3/Nm³ measuring range.). The ground tire rubber was obtained from Rubber Recycling, Pelt, Belgium in a 0.5 – 1 mm mesh size, which was later cryogenically powdered in-house using a Fritsch Pulverisette 14 equipped with a 0.3 mm mesh size sieve ring to 0.3 mm particle size in order to increase the surface area. The crumb rubber originated from passenger tires in a mixed streams of models and brands. Synthos Sprintan 3402 was obtained from Synthos Schkopau GMBH in Germany. Oxygen (>99.5%; Air liquide), Chloroform (>99,8%; Chem-Lab), Methylene chloride (>99.8%; Fischer Scientific), Methanol (HPLC grade, Chem-Lab), Ethanol (99%; Fischer Scientific), Norit Activated charcoal CN 1 (Acros Organics), potassium iodide (99%; Acros Organics) and triethylamine (99%; Acros Organics) were used as purchased.

ANALYTICAL METHODS

A Bruker alpha II FTIR spectrometer equipped with a diamond crystal was used for FTIR analysis (24 scans, 600-4000 cm-1, 4 µm resolution).

A Shimadzu GC-2010 gas chromatograph equipped with a Shimadzu QP2010S mass spectrometer and Phenomenex Zebron ZB-5ms column was used for GC-MS analysis with a 1:10 split ratio. The temperature was equilibrated at 30 °C and held for 2 min. The temperature was ramped to 220 °C at a rate of 10 °C/min and held for an additional 5 min. Finally, the temperature was further increased to 320 °C at a rate of 10 °C/min and held for 20 min.
Quantification was performed using gas chromatography (agilent G7820A) coupled with flame ionization detection (GC-FID) equipped with a Phenomenex Zebron ZB-5ms column ran in splitless injection mode. The temperature was equilibrated at 50 °C and held for 2 min. The temperature was ramped to 220 °C at a rate of 10 °C/min and held for an additional 5 min. Finally, the temperature was further increased to 320 °C at a rate of 10 °C/min and held for 20 min.

The obtained acids were esterified in triplicate to methyl esters over Amberlyst 35 and molecular sieves in methanol at 65°C overnight. Methyl decanoate was added as internal standard.

Calibration curves were constructed for methyl ester-derivatized samples of levulinic (Rt = 7.3 min), succinic ($Rt_{dimethyl}$ = 7.8 min, $Rt_{monomethyl}$ = 9.5 min), Stearic (Rt = 19 min) and Palmitic Acid (Rt = 21.5 min) in the range of 50 - 2000 ppm with methyl decanoate (Rt = 12 min) as internal standard.

Thermogravimetric analysis was performed on a TA Instruments TGA Q5000 using Platinum – HT type pans. The product was initially heated up under inert atmosphere (nitrogen; Air Liquide; 99.999%) from 50 to 600 °C (heating rate of 10 °C/min) to pyrolyze any organic carbon. Then, the oven was cooled to 300 °C, followed by the switching from nitrogen to air atmosphere. The product was then heated again until 800 °C (heating rate of 10 °C/min) to oxidize any formed pyrolytic carbon as well as any already present graphitic carbon to carbon black. ICP-OES analysis was performed (externally by ACC/IUMAT of Hasselt University) in duplo on two samples after acid digestion (HCl/HNO3/HBF4). ICP-OES was performed on a Perkin-Elmer Optima 8300. ED-XRF analysis was performed (externally by ACC/IUMAT of Hasselt University) on a Shimadzu EDX 8100.

EXPERIMENTAL RESULTS

Table S1: Experimental data of ozonolysis in DCM (1-7), DCM/10v% ethanol (8-16) and $CHCl_3$ (17-24)

Reaction	starting mass rubber (g)	Mass crude product (g)	Mass Residue (g)
DCM-1	5.1900	2.9639	3.1153
DCM-2	4.9980	2.5437	2.9571
DCM-3	1.0109	0.6038	0.5395
DCM-4	1.1127	0.6759	0.5044
DCM-5	1.0062	0.6017	0.4858
DCM-6	1.0500	0.6624	0.5545
DCM-7	1.0286	0.6166	0.4240
DCM-EtOH-8	1.1820	0.8814	0.3999
DCM-EtOH-9	5.1220	4.5041	1.7231
DCM-EtOH-10	5.7103	4.4994	2.2014
DCM-EtOH-11	5.6146	3.5726	2.6834
DCM-EtOH-12	1.0861	0.8122	0.5385
DCM-EtOH-13	1.0351	0.8258	0.5269
DCM-EtOH-14	1.0467	0.8113	0.5291
DCM-EtOH-15	1.0516	0.7481	0.5831
DCM-EtOH-16	0.9959	0.7631	0.4773
CHCl3-17	1.0020	0.7925	0.3587
CHCl3-18	1.0536	0.765	0.3797
CHCl3-19	1.0083	0.7396	0.3405
CHCl3-20	0.9987	0.6596	0.3536
CHCl3-21	1.0026	0.7076	0.3400
CHCl3-22	1.0365	0.7339	0.2951
CHCl3-23	1.002	0.817	0.3566
CHCl3-24	1.021	0.794	0.3768

ANALYSIS RESULTS

FOURIER TRANSFORM INFRARED SPECTROSCOPY

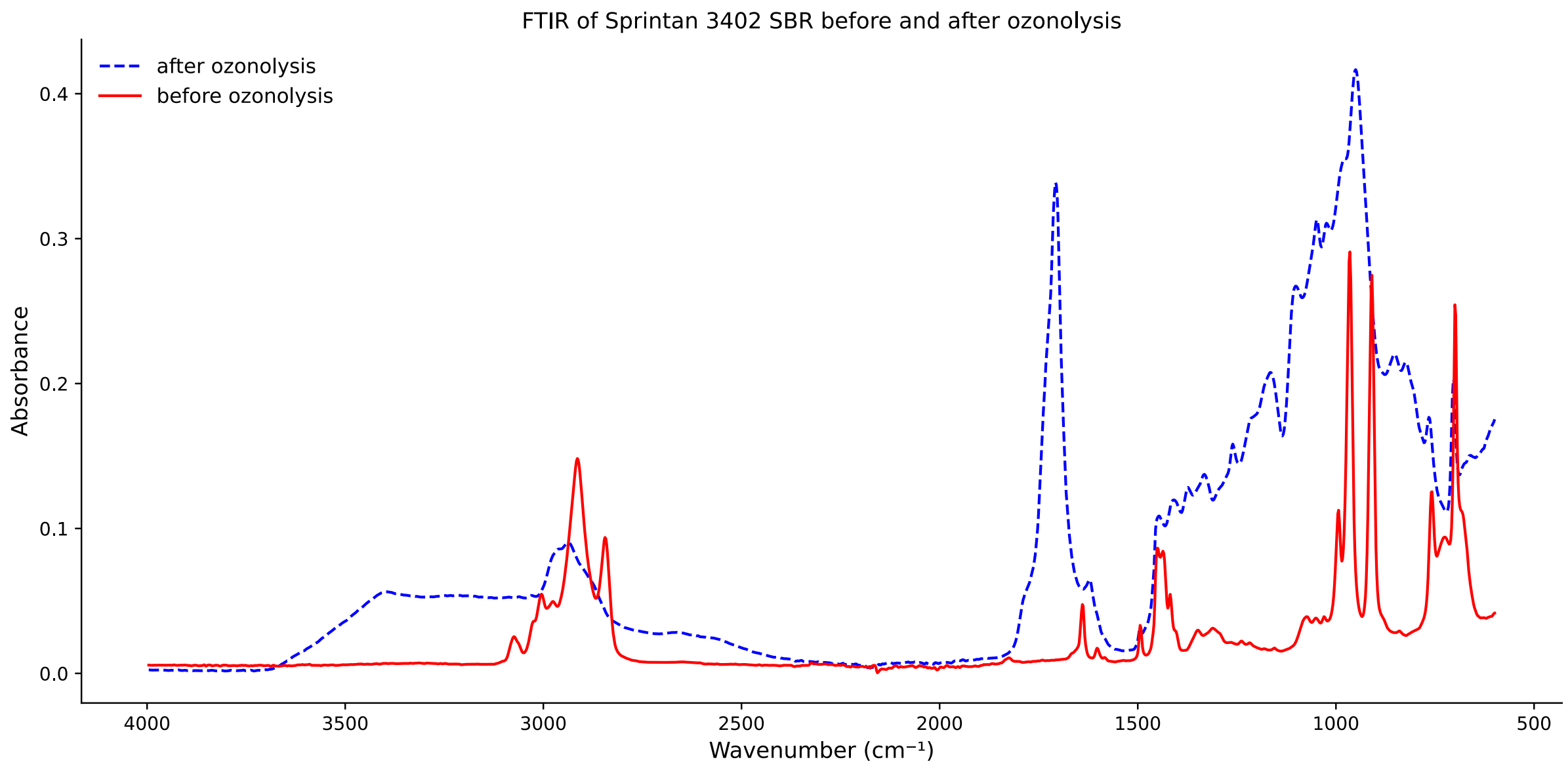

Figure S1: FTIR spectra of Sprintan 3402 SBR before (red, solid), and after (blue, dashed) ozonolysis

^{1}H-NUCLEAR MAGNETIC RESONANCE SPECTROSCOPY

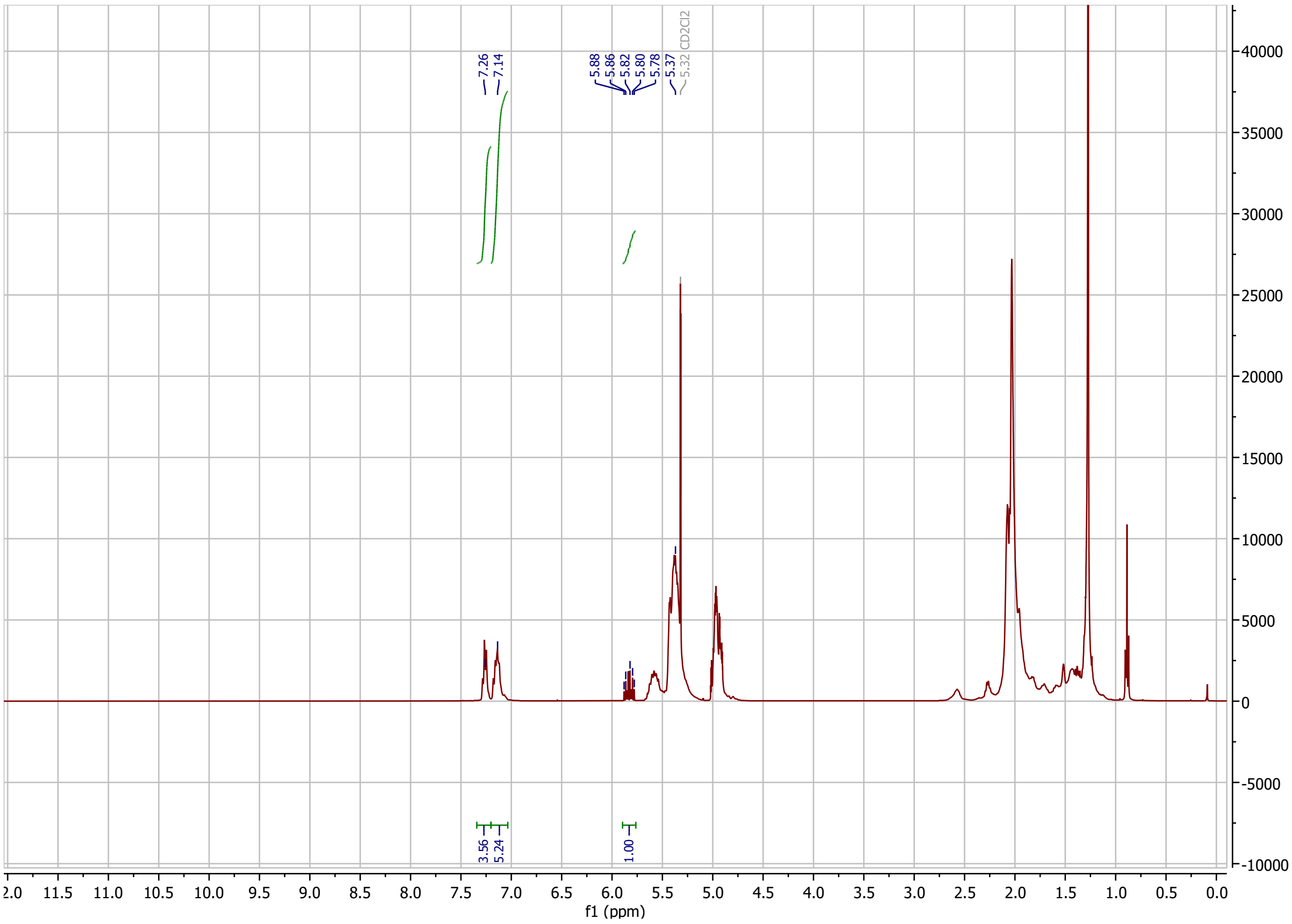

Figure S2: ^{1}H-NMR spectrum of Sprintan 3402 SBR prior to ozonolysis in $CDCl_2$ with 1-docosane added as internal standard

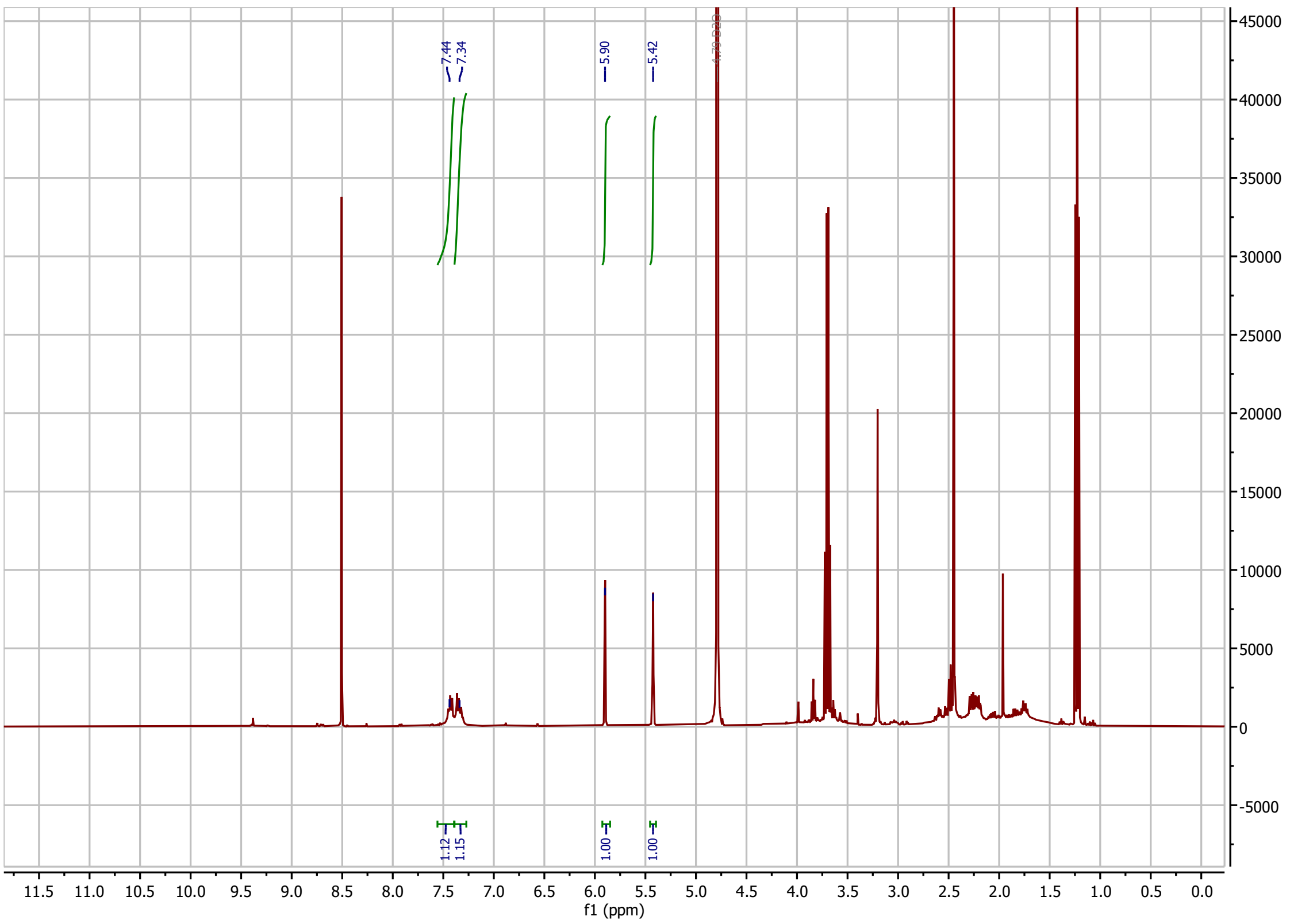

Figure S3: ¹H-NMR spectrum of Sprintan 3402 SBR following ozonolysis in NaOD/D_2O with Sodium Itaconate added as internal standard

Table S2: ¹H-NMR quantification data of Sprintan 3402 SBR prior to ozonolysis in $CDCl_2$ (internal standard = 1-docosene) and post ozonolysis in NaOD/D_2O (internal standard = sodium itaconate)

	Mass analyte (g)	Mass internal standard (g)	Molar mass standard (g/mol)	Moles internal standard (moles)	Integral analyte {ppm}	Integral internal standard {ppm}	Nuclei ratio of aromatic : standard	Mass styrene equiv. (g)	Wt% sample
Sprintan 3402 prior to ozonolysis	0.0168	0.0037	308.594	1.21 x 10^{-5}	8.8 {δ 7.26, 7.14)	1 {5.82}	5:1	0.0022	13
Sprintan 3402 post ozonolysis	0.0243	0.0050	130.099	3.85 x 10^{-5}	2.27 {δ 7.44, 7.34}	1 {5.90}	5:1	0.0018	7.5

THERMOGRAVIMETRIC ANALYSIS

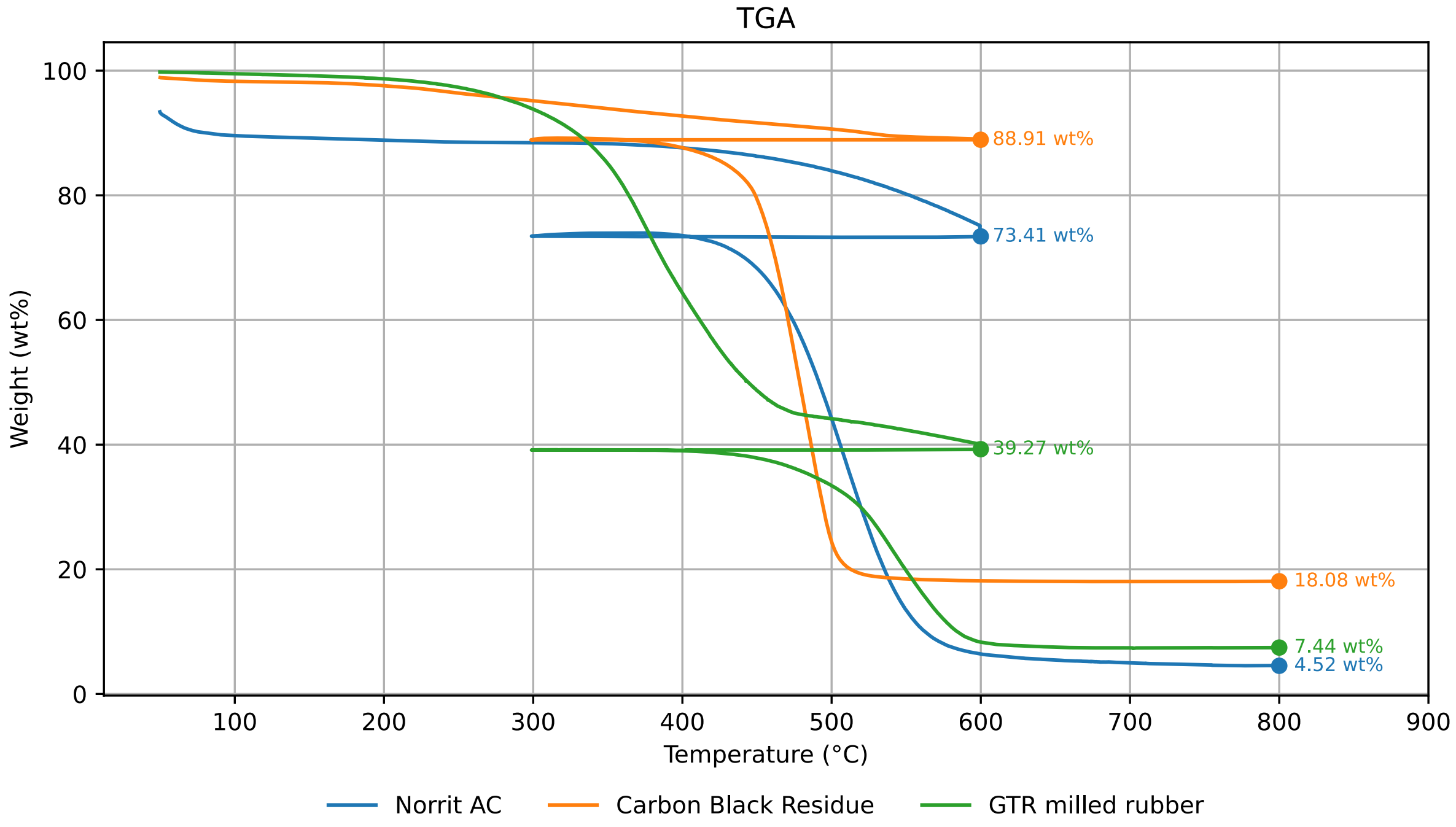

Figure S4: TGA plot of activated charcoal (Norit CN1) (blue), obtained carbon black (orange) and ground tire rubber (green) as well as the remaining wt% at 600 °C after pyrolysis under N_2 atmosphere and at 800 °C after combustion under air

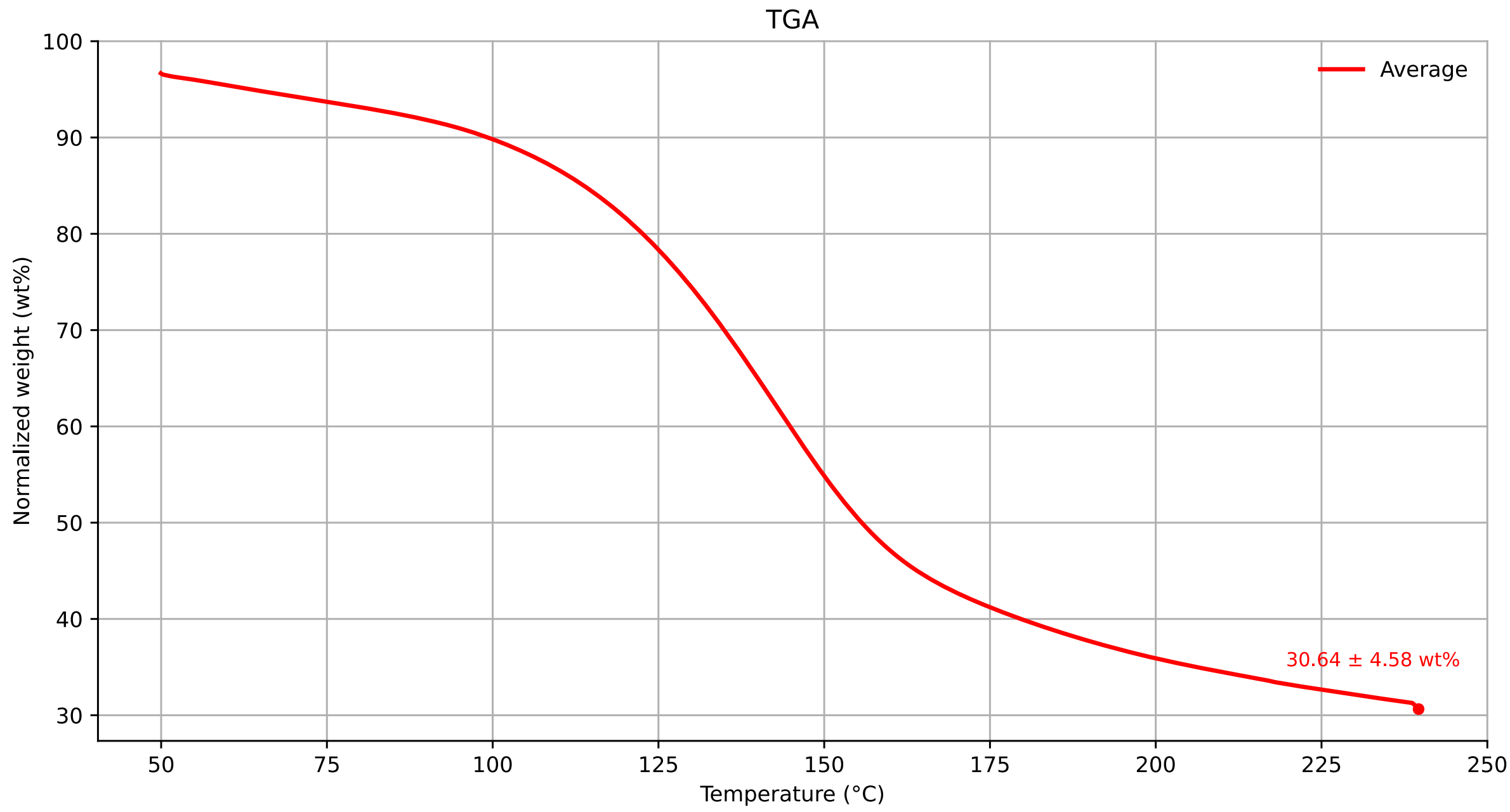

Figure S5: TGA plot of heating loss of average of 3 ozonolysis-hydrolysis products heated to 240 °C under 60 mL/min N_2 atmosphere

INDUCTIVE COUPLED PLASMA – OPTICAL EMISSION SPECTROMETRY ANALYSIS

Table S3: ICP-OES result of mineral fraction of ozonolysis carbon black

Element	ICP-OES (wt%) (n=2)	Oxide Equivalent (wt%)	ICP-OES (mg/kg)	Oxide Equivalent (mg/kg)
Zn	1.43 ± 0.02	1.78 (ZnO)		
Al	1.25 ± 0.02	2.36 (Al_2O_3)		
Ca	1.96 ± 0.04	2.74 (CaO)		
K	0.65 ± 0.01	0.78 (K_2O)		
Mg	0.27 ± 0. 01	0.45 (MgO)		
Na	0.32 ± 0.01	0.43 (Na_2O)		
Fe	5.29 ± 0.02	7.56 (Fe_2O_3)		
S	1.74 ± 0.02	4.35 (SO_3)		
Cu	1.70 ± 0.05	2.12 (CuO)		
P	0.49 ± 0.02	1.13 (P_2O_5)		
Ba	0.99 ± 0.00	1.10 (BaO)		
Si	31.8 ± 0.1	68.0 (SiO_2)		
Mn			64.7 ± 1.1	83.6 (MnO)
Ni			24.5 ± 1.0	31.2 (NiO)
Pb			421 ± 4	454 (PbO)
Sr			51.2 ± 1.1	60.5 (SrO)
Co			250 ± 4	318 (CoO)
Cr			62.3 ± 1.6	91.1 (Cr_2O_3)
Li			47.3 ± 0	102 (Li_2O)
Bi			< 33	
Cd			< 3	
Ag			< 3	

GAS CHROMATOGRAPHY – MASS SPECTROMETRY ANALYSIS

GAS CHROMATOGRAMS

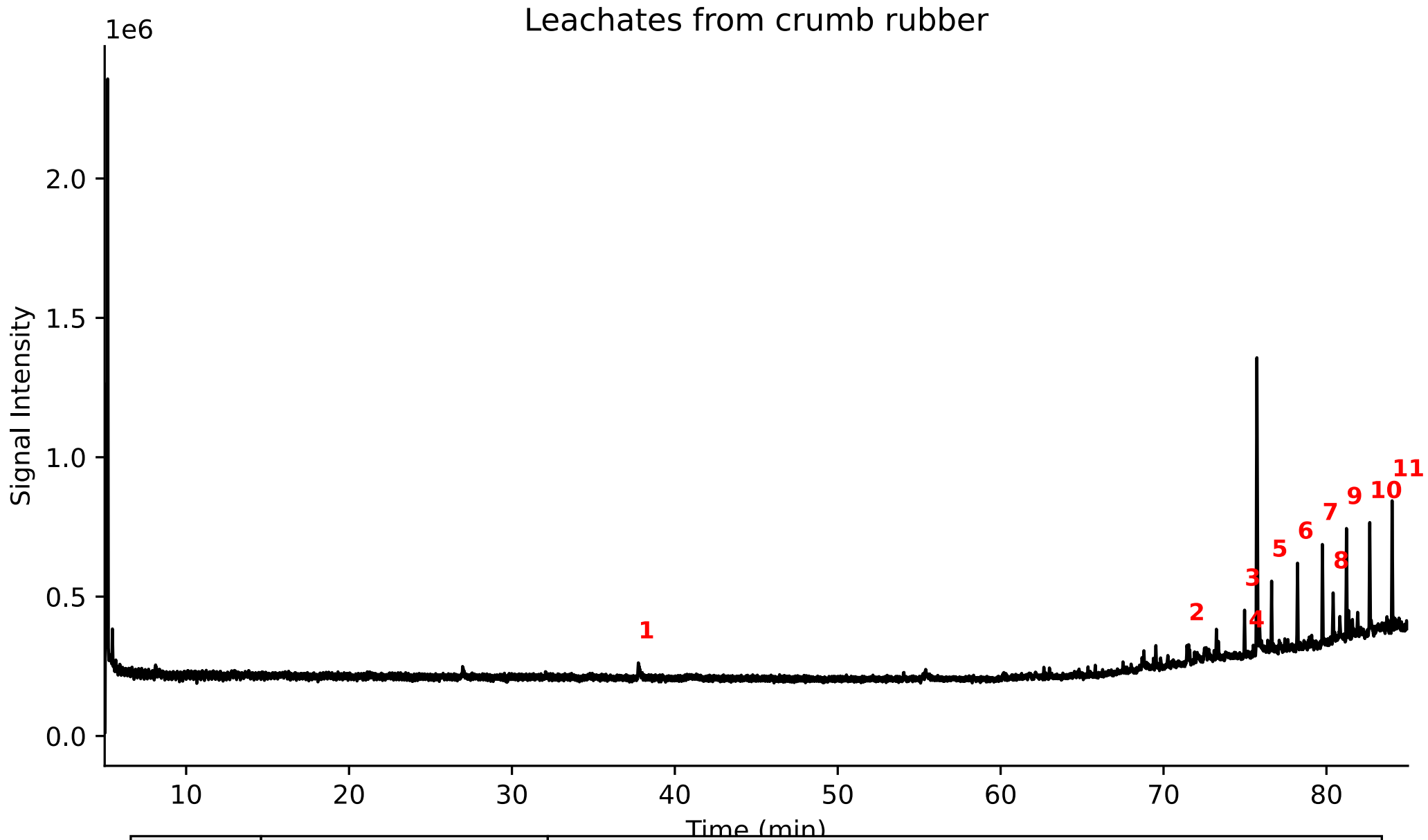

No.	Retention time (min)	Compound
1	37.76	Benzothiazole
2	71.55	Benzene, [(3-phenyl-2-propenyl)thio]-
3	74.97	Tricosane
4	75.22	1,4-Benzenediamine, N-(1,3-dimethylbutyl)-N'-phenyl-
5	76.63	Tetracosane
6	78.23	Pentacosane
7	79.75	Hexacosane
8	80.41	4,4'-(p-Phenylene)diisopropylidene)diphenol
9	81.23	Heptacosane
10	82.66	Octacosane
11	84.03	Nonacosane

Figure S6: GC-MS result of leachates from crumb rubber

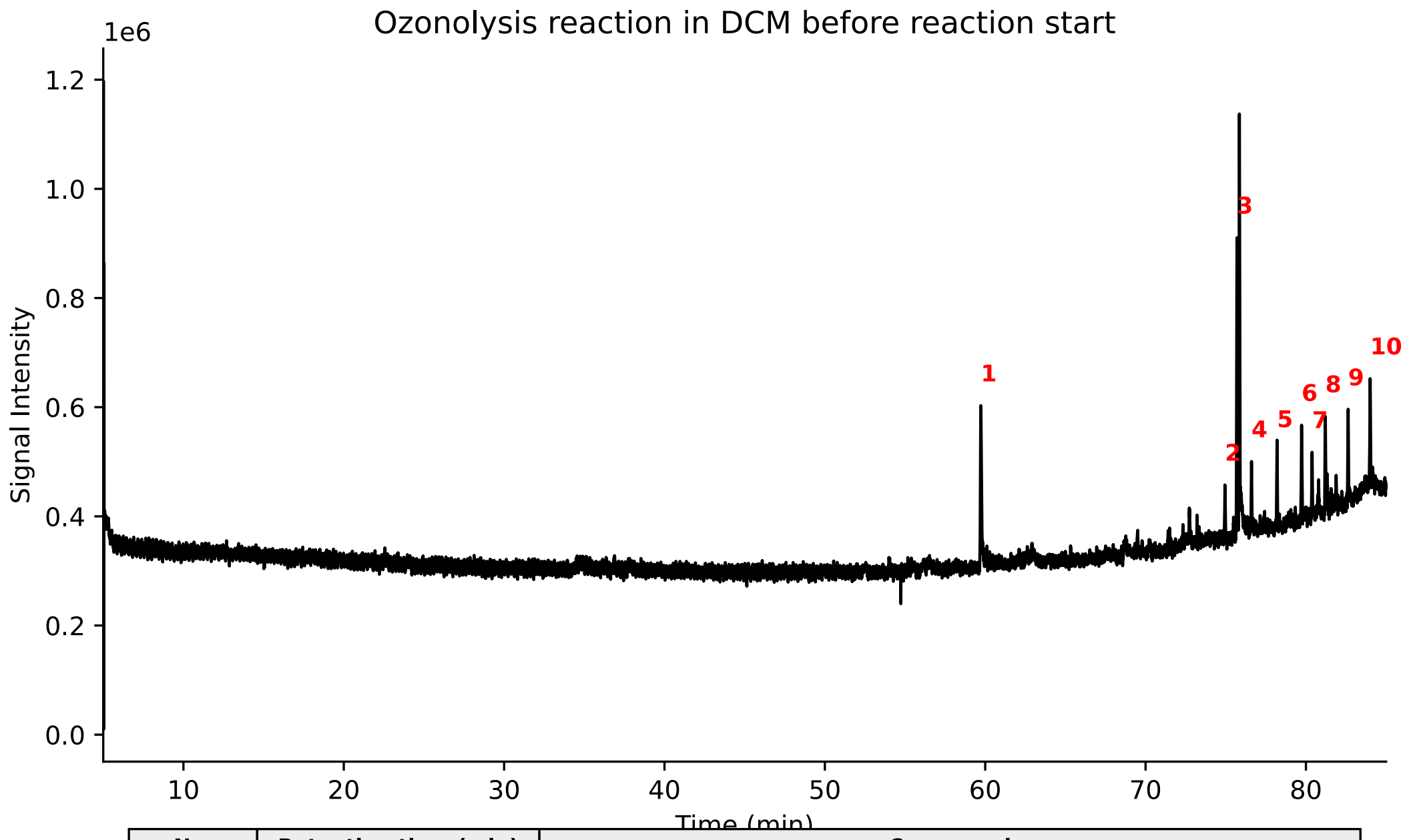

No.	Retention time (min)	Compound
1	59.73	Diethyl Phtalate
2	74.95	Tricosane
3	75.70	1,4-Benzenediamine, N-(1,3-dimethylbutyl)-N'-phenyl-
4	76.61	Tetracosane
5	78.19	Pentacosane
6	79.73	Hexacosane
7	80.39	4,4'-(p-Phenylene)diisopropylidene)diphenol
8	81.21	Heptacosane
9	82.63	Octacosane
10	84.01	Nonacosane

Figure S7: GC-MS result of ozonolysis reaction in DCM after swelling overnight, before reaction start

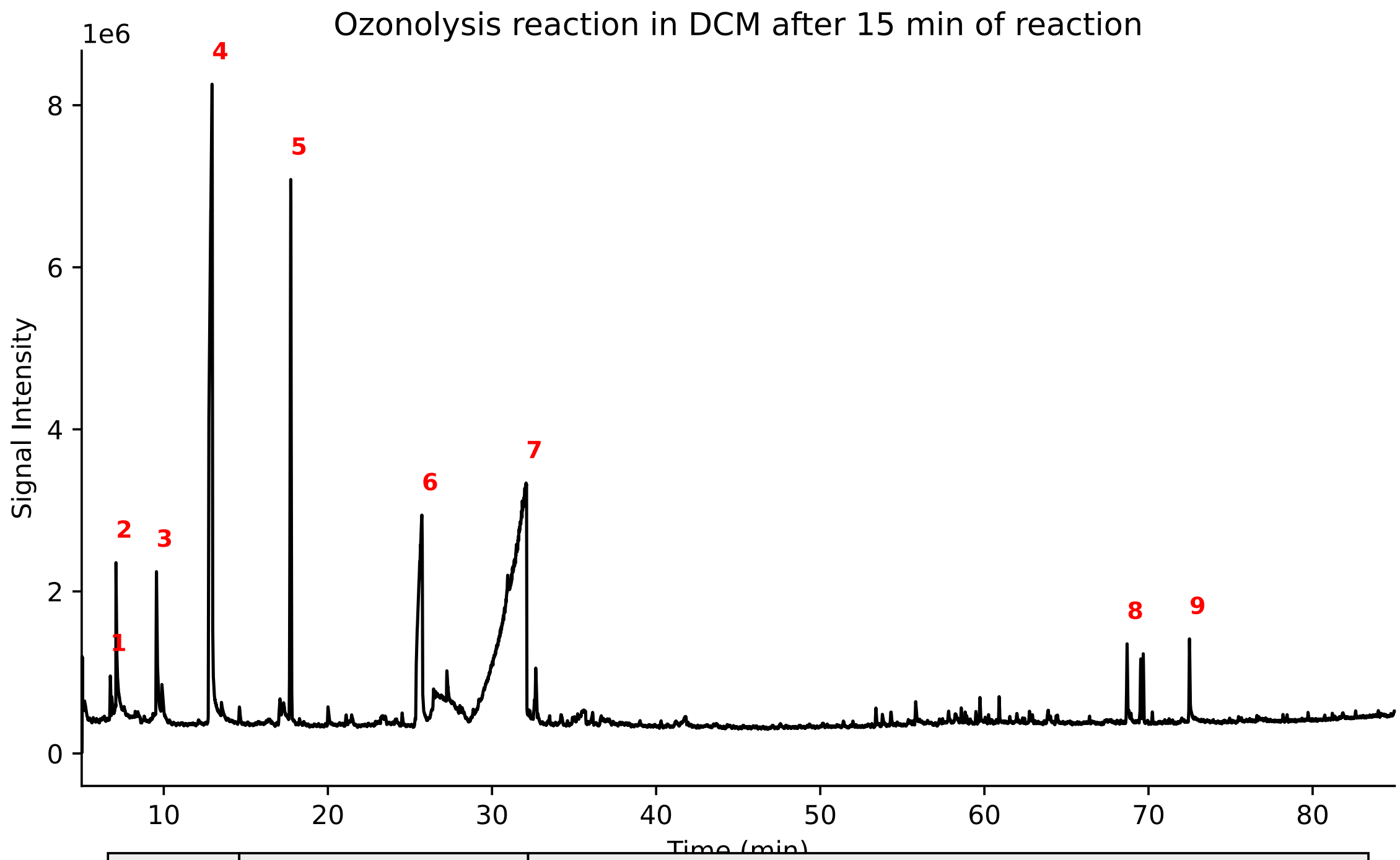

No.	Retention time (min)	Compound
1	6.75	Ethane, 1,1,2-trichloro-
2	7.09	Succindialdehyde
3	9.55	4-Chloro-2-butanone
4	12.94	4-Oxopentanal
5	17.74	Ethane, 1,1,2,2-tetrachloro
6	25.73	Succinic Anhydride
7	32.08	Pentanoic Acid, 4-oxo-
8	68.69	n-Hexadecanoic acid
9	72.50	Octadecanoic acid

Figure S8: GC-MS result of ozonolysis reaction in DCM after swelling overnight, after 15 min of reaction

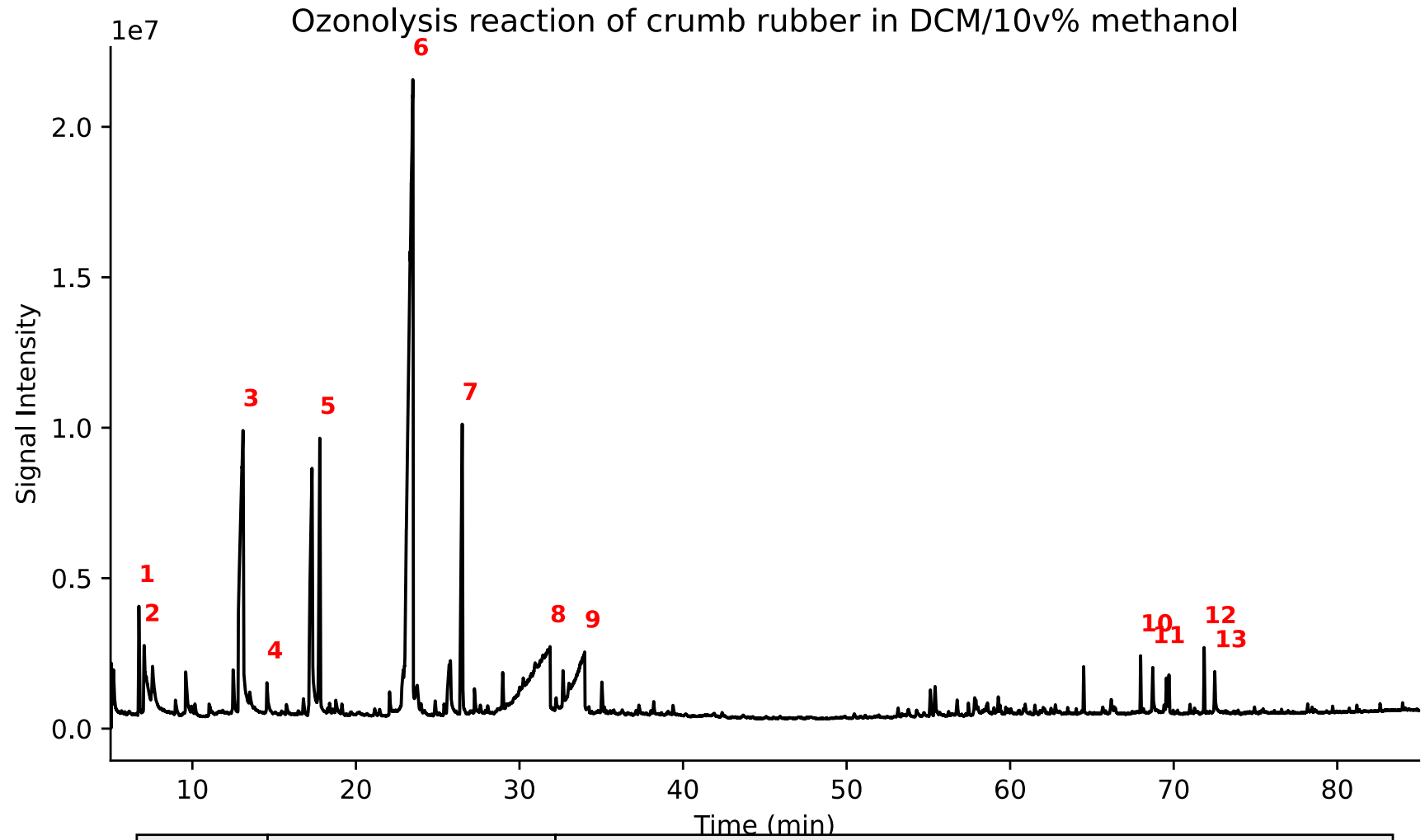

No.	Retention time (min)	Compound
1	6.73	Ethane, 1,1,2-trichloro-
2	7.05	Ethanol, 2,2-dichloro
3	13.10	4-oxopentanal
4	14.56	Furan, Tetrahydro-2,5-dimethoxy-
5	17.79	Ethane, 1,1,2,2-tetrachloro-
6	23.48	Pentanoic acid, 4-oxo-, methyl ester
7	26.50	Butanedioic acid, dimethyl ester
8	31.87	Pentanoic acid, 4-oxo-
9	33.98	Butanedioic acid, monomethyl ester
10	67.97	Hexadecanoic acid, methyl ester
11	68.72	Hexadecanoic acid
12	71.86	Octadecanoic acid, methyl ester
13	72.51	Octadecanoic acid

Figure S9: GC-MS result of ozonolysis reaction of crumb rubber in DCM/10v% methanol

MASS SPECTROMETRY COMPOUND TABLES

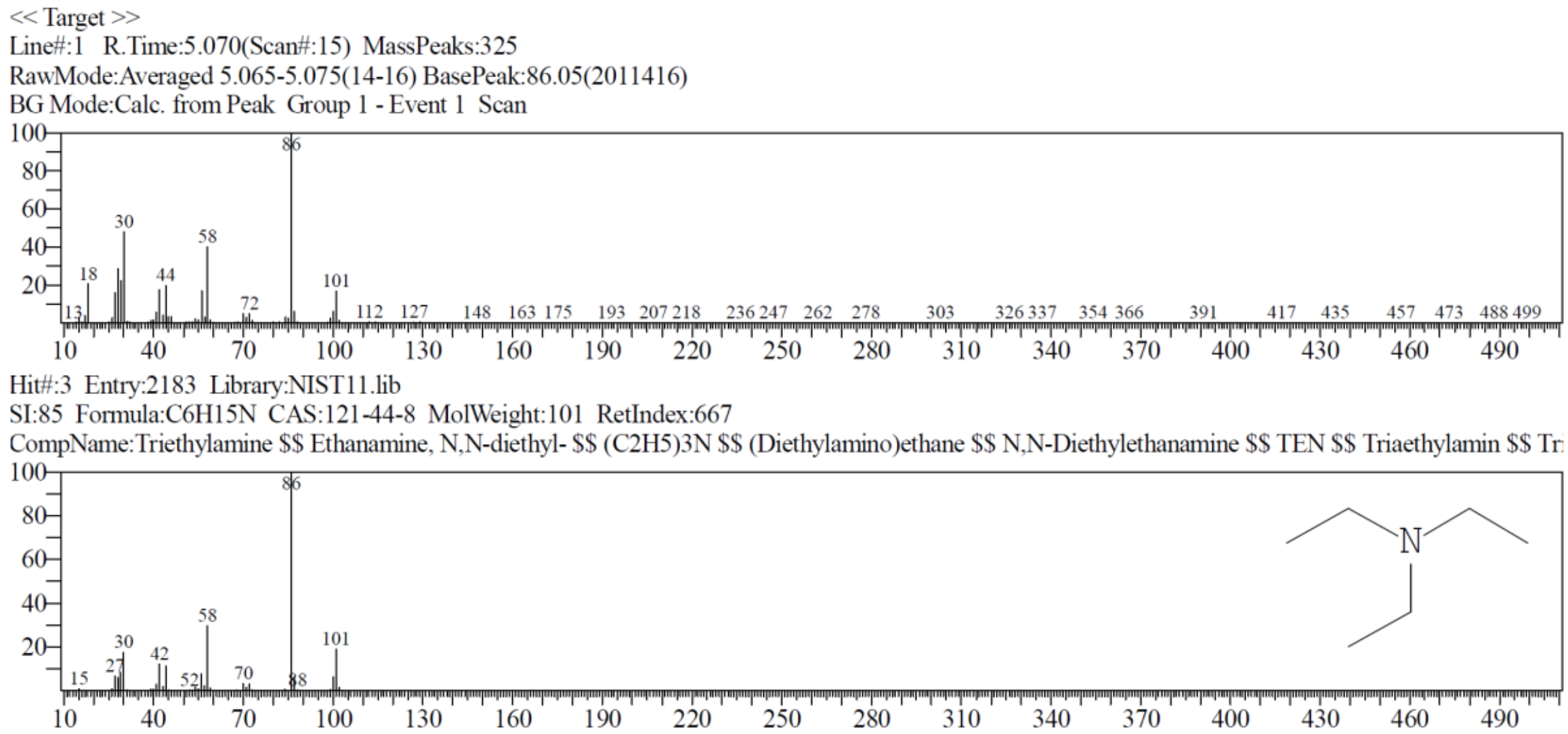

Figure S10: Mass spectrum of triethylamine and library reference spectrum

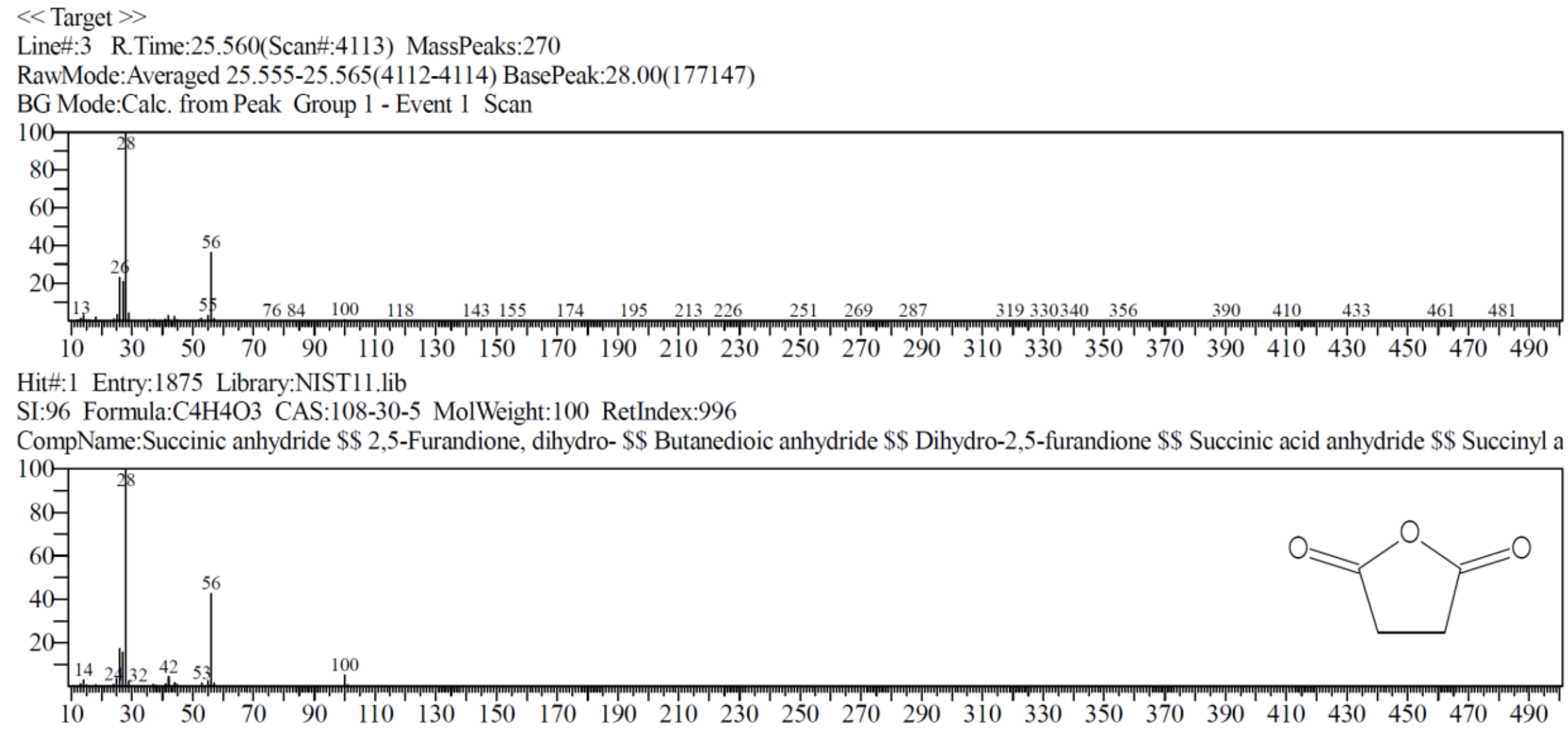

Figure S11: Mass spectrum of succinic anhydride and library reference spectrum

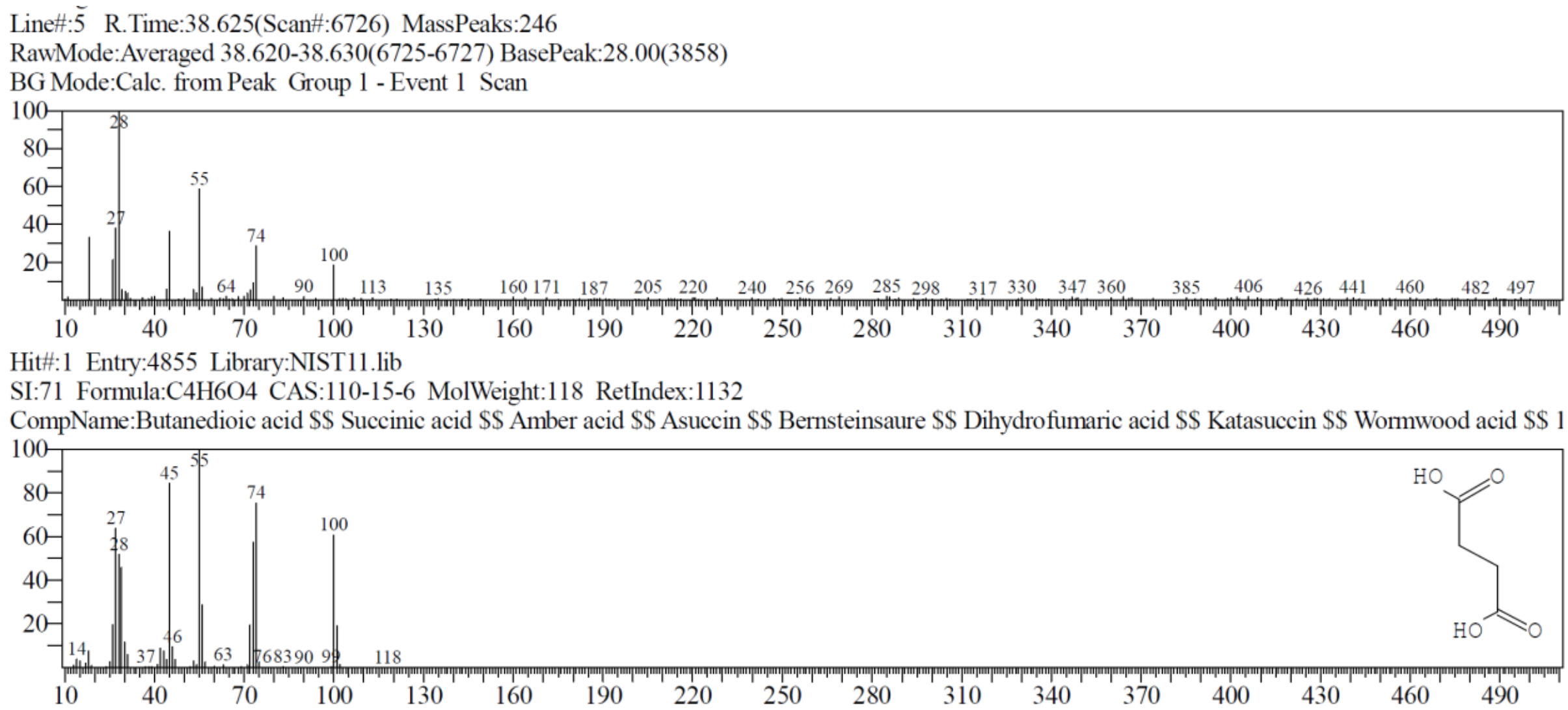

Figure S12: Mass spectrum of succinic acid and library reference spectrum

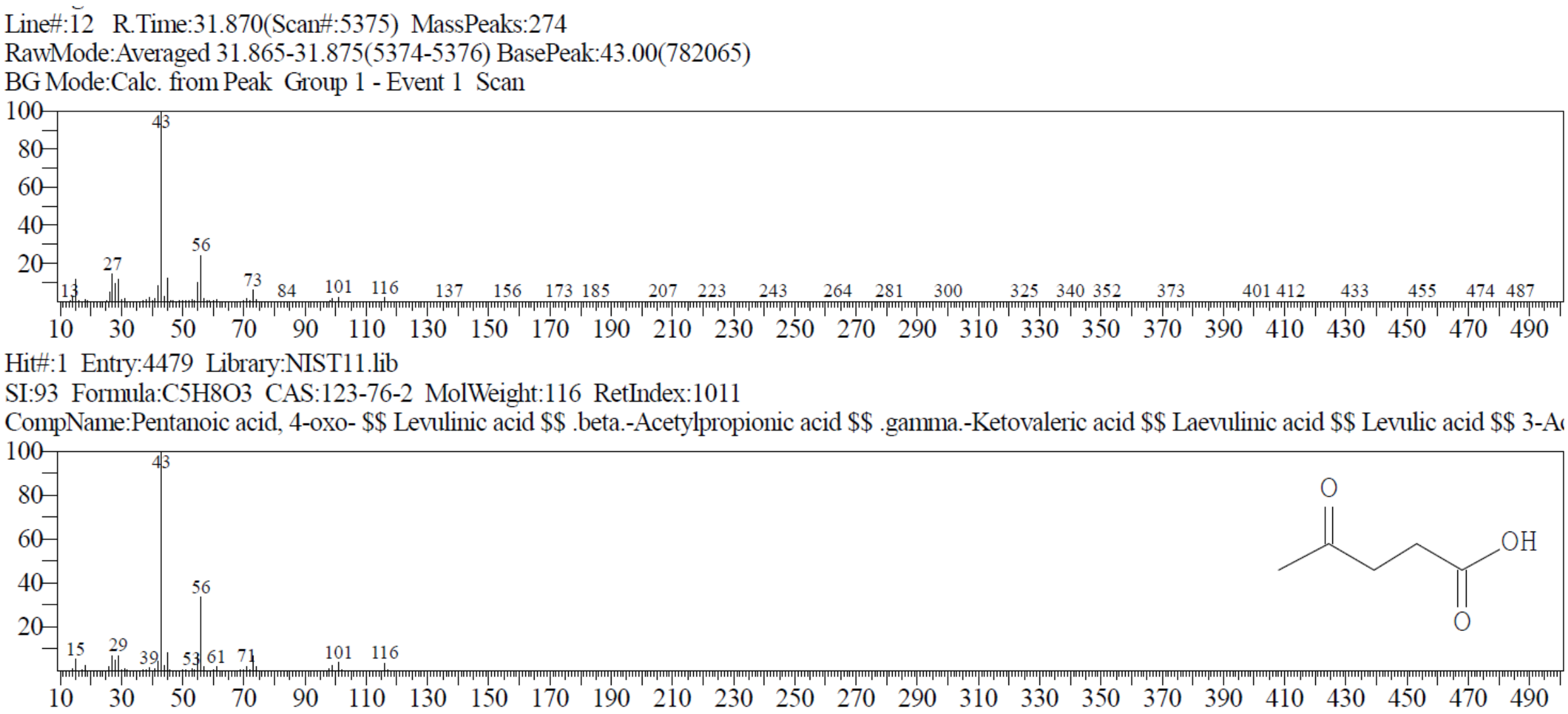

Figure S13: Mass spectrum of levulinic acid and library reference spectrum

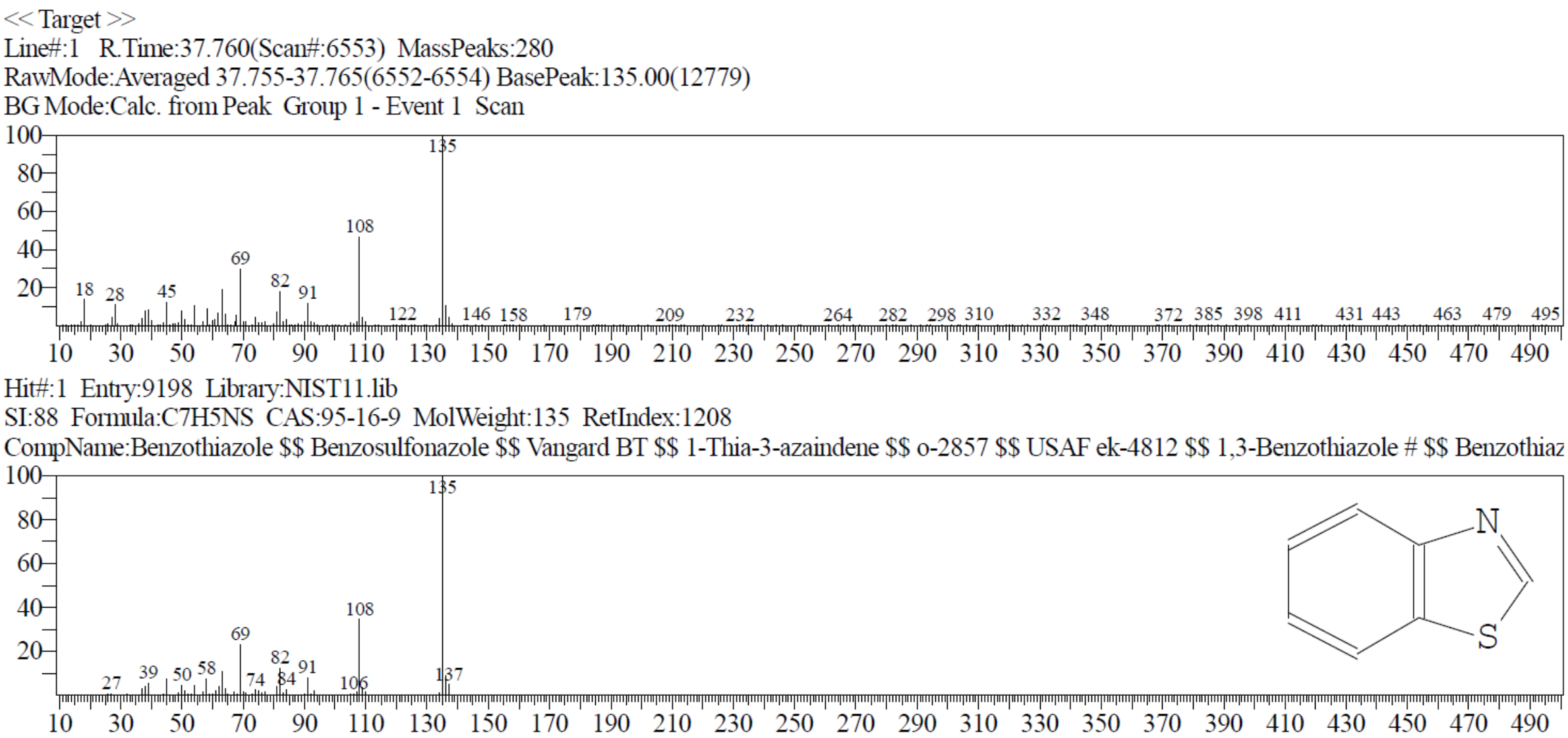

Figure S14: Mass spectrum of benzothiazole antiozonant and library reference spectrum

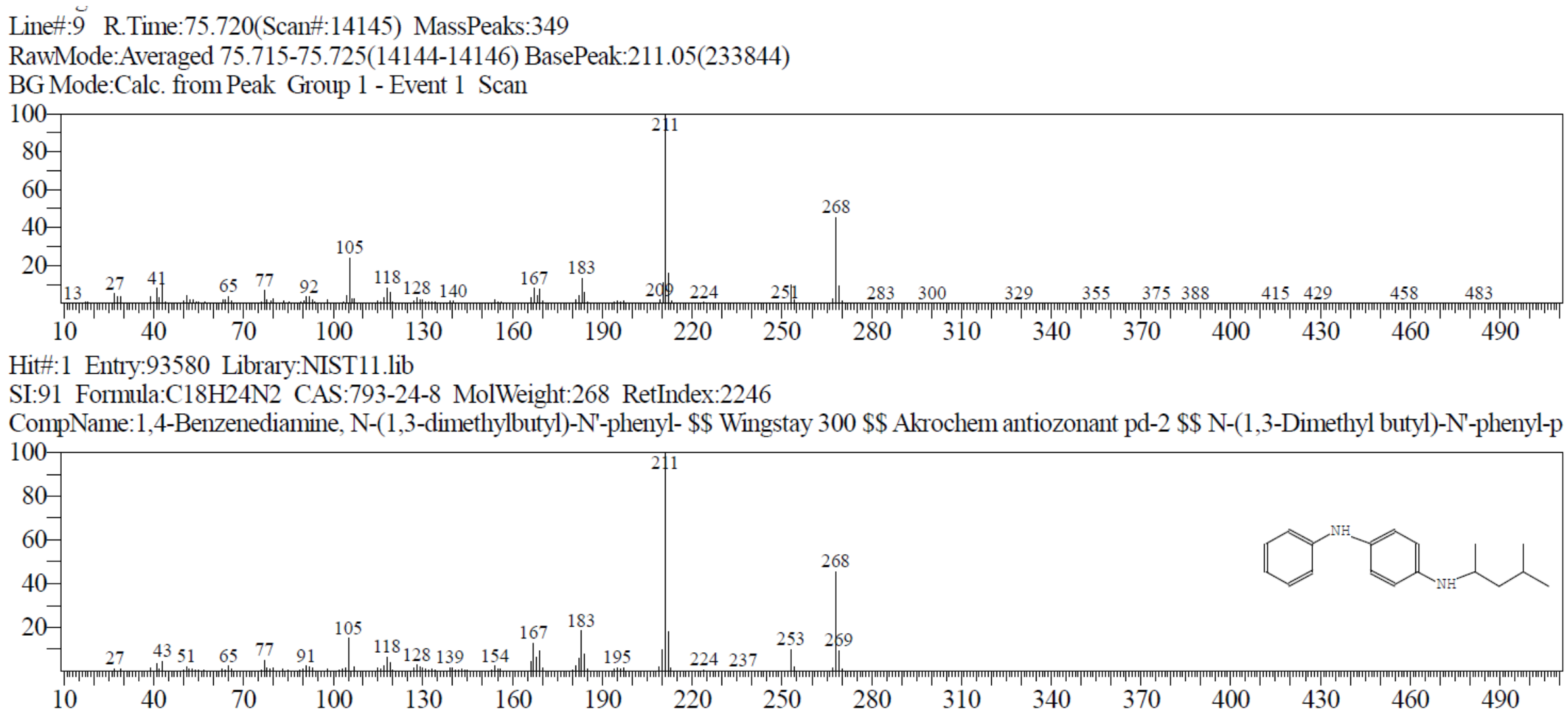

Figure S15: Mass spectrum of N1-(4-methylpentan-2-yl)-N4-phenylbenzene-1,4-diamine - antiozonant and library reference spectrum

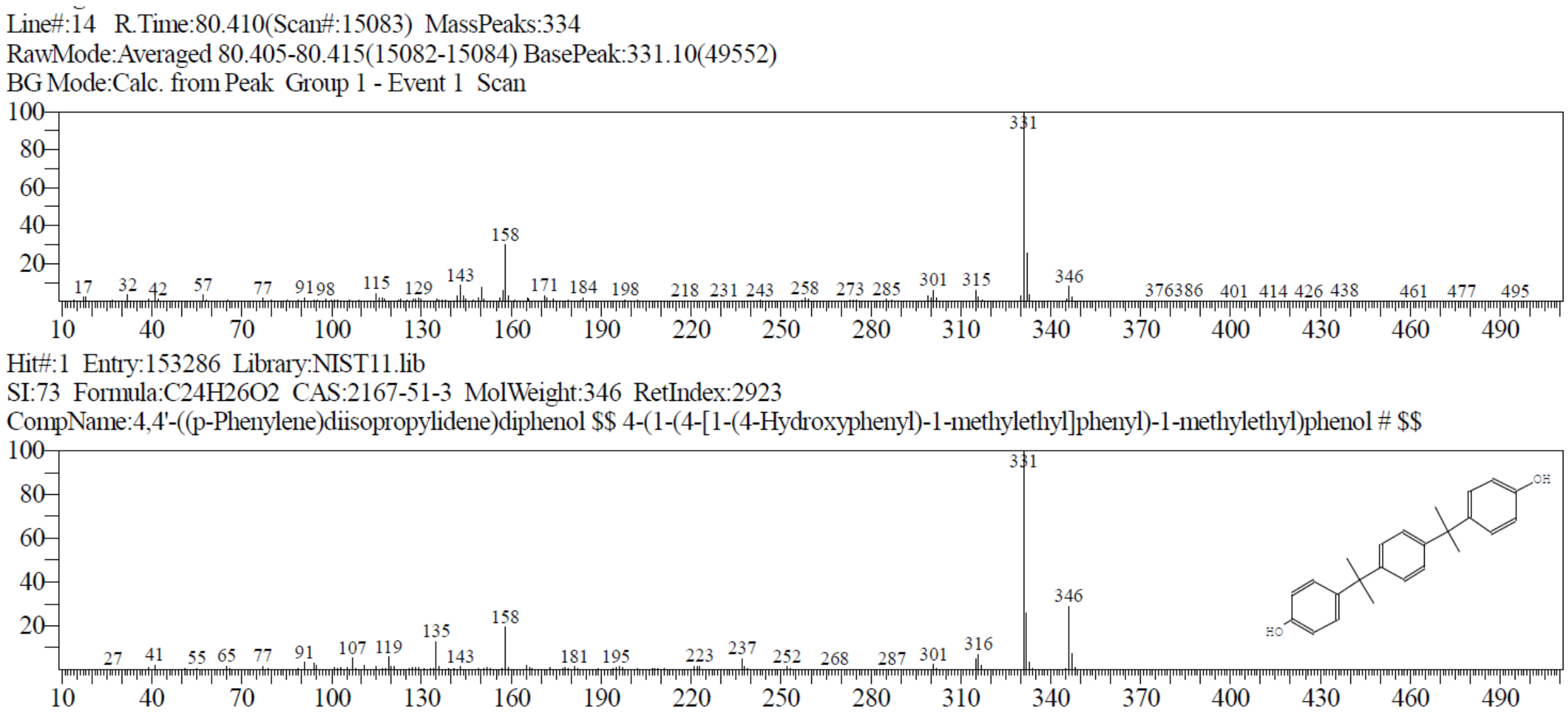

Figure S16: Mass spectrum of 4,4'-((p-phenylene)diisopropylidene)diphenol - antiozonant and library reference spectrum

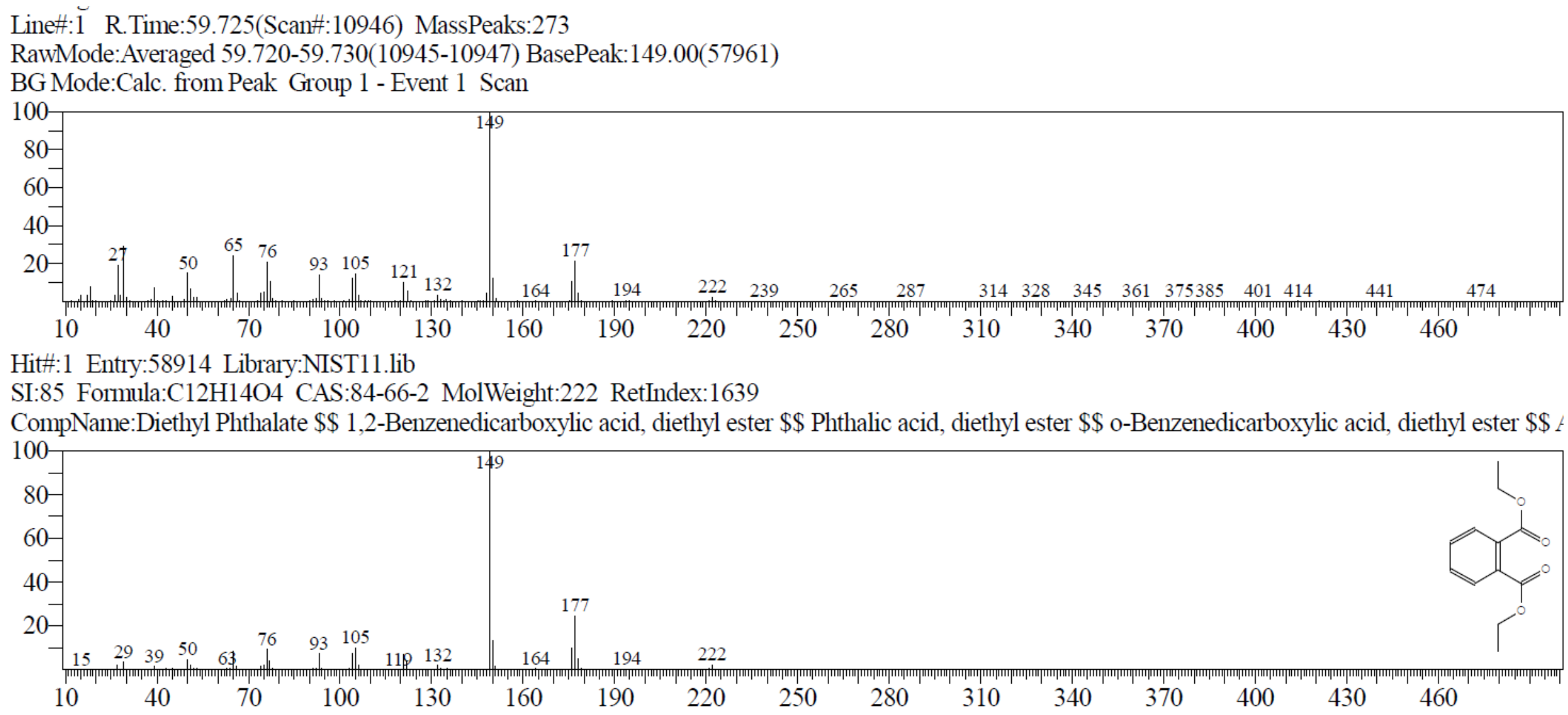

Figure S17: Mass spectrum of diethyl phthalate - plasticizer and library reference spectrum

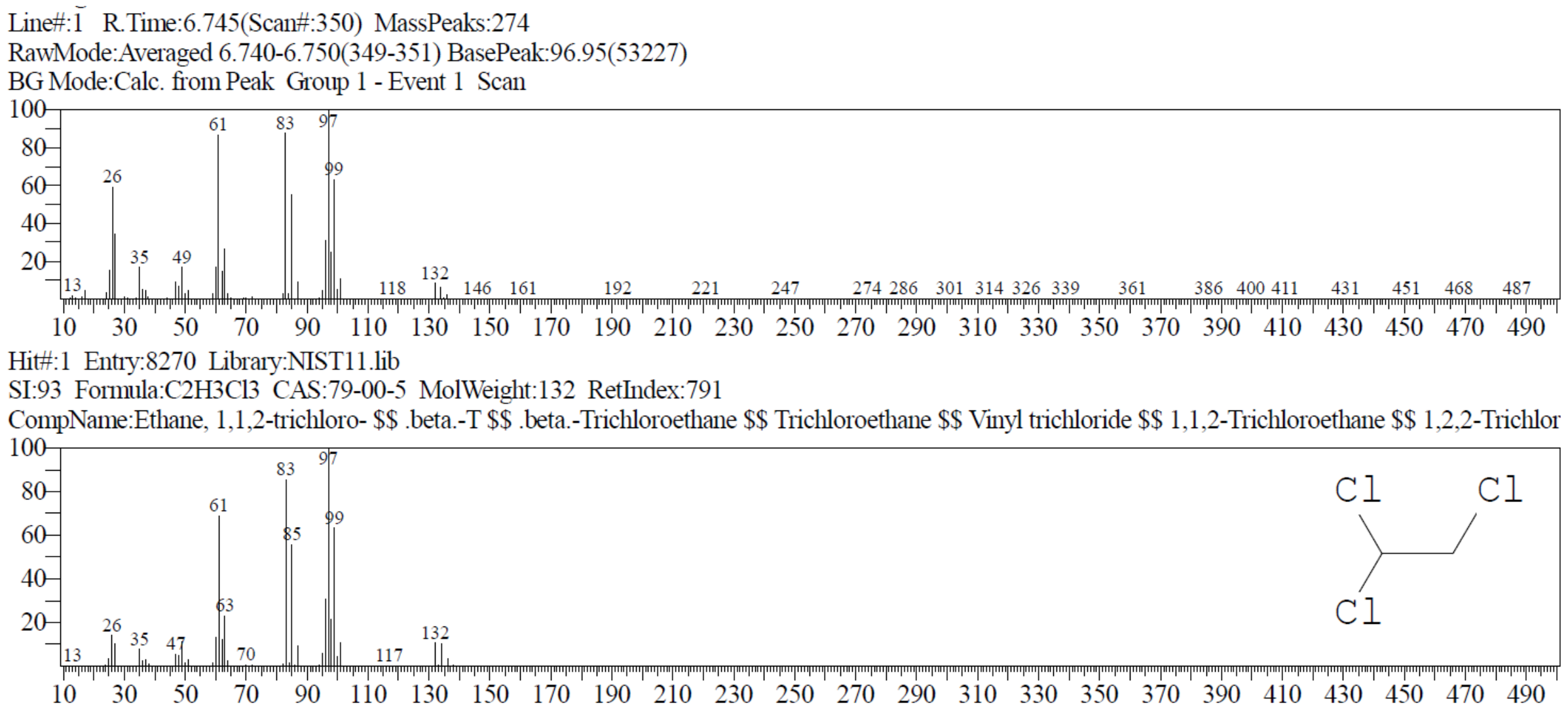

Figure S18: Mass spectrum of 1,1,2-trichloro-ethane - DCM side product and library reference spectrum

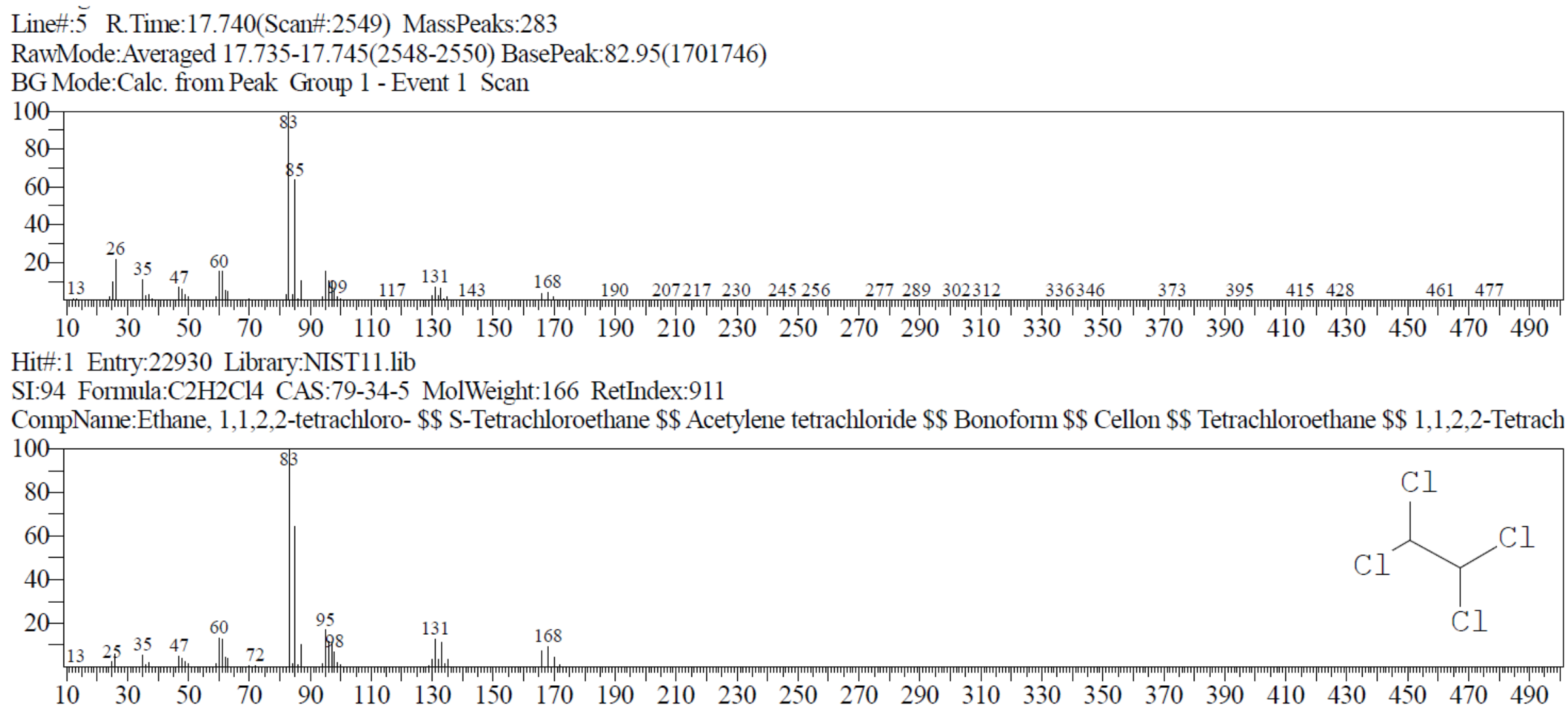

Figure S19: Mass spectrum of 1,1,2,2-tetrachloro-ethane - DCM side product and library reference spectrum

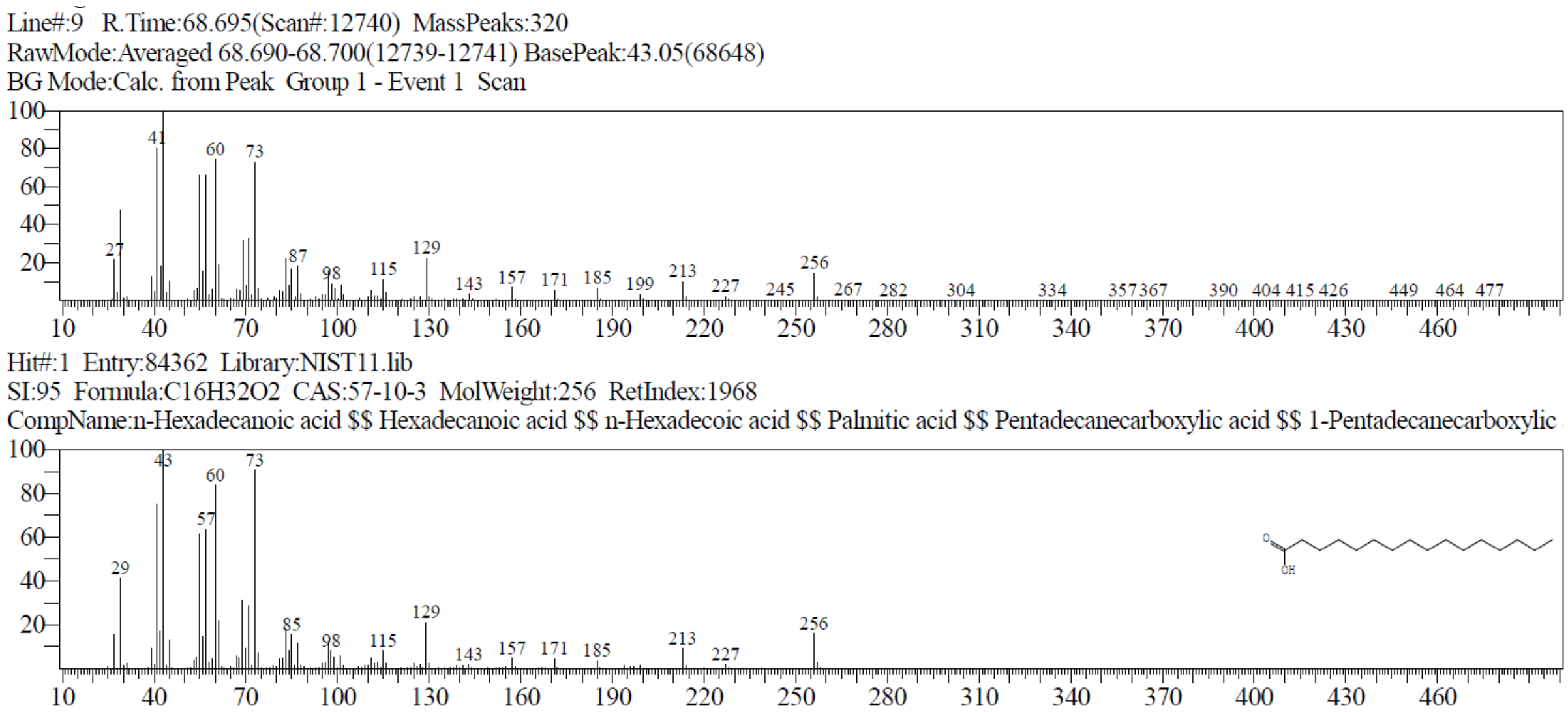

Figure S20: Mass spectrum of n-hexadecanoic acid - oxidized physical anti-ozonant wax and library reference spectrum

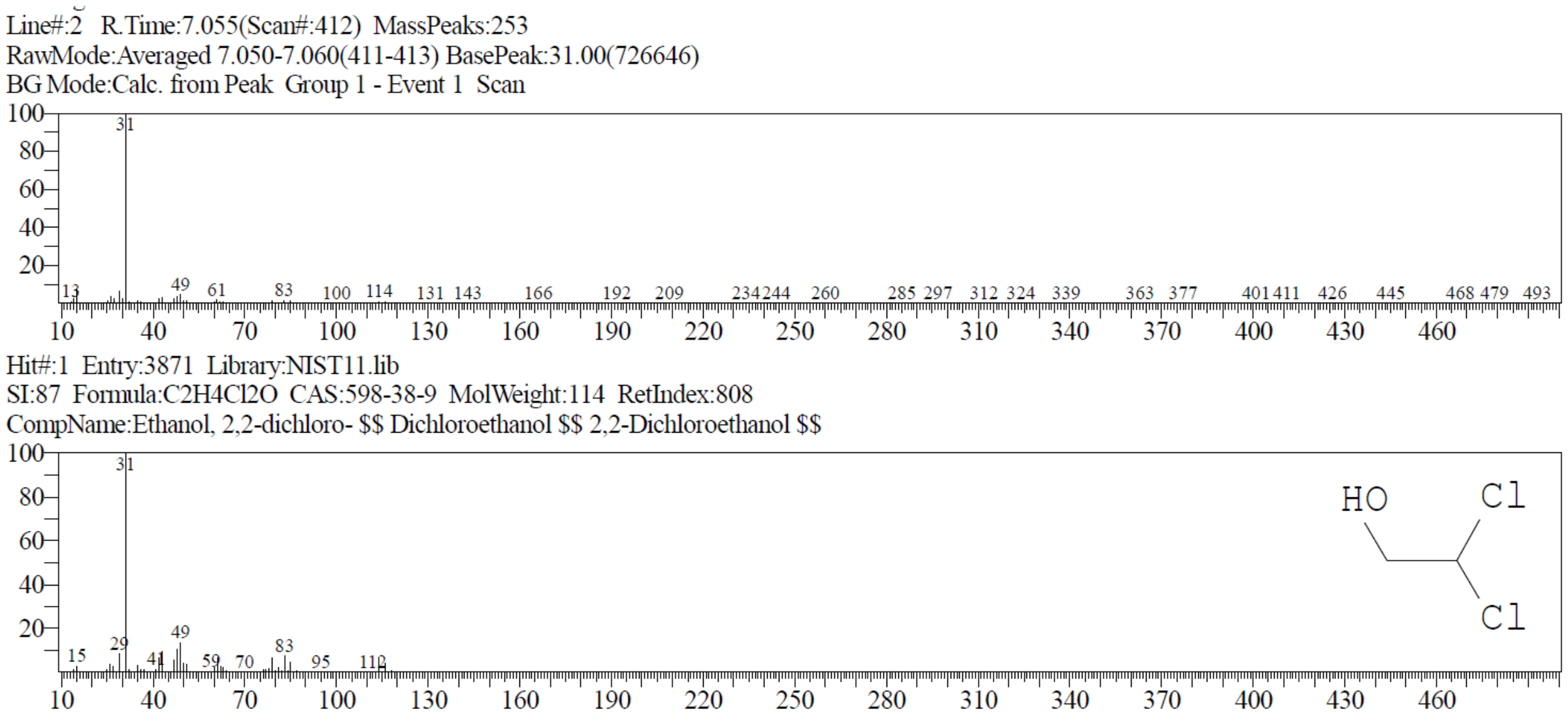

Figure S21: Mass spectrum of 2,2-dichloroethanol - DCM and methanol side product and library reference spectrum

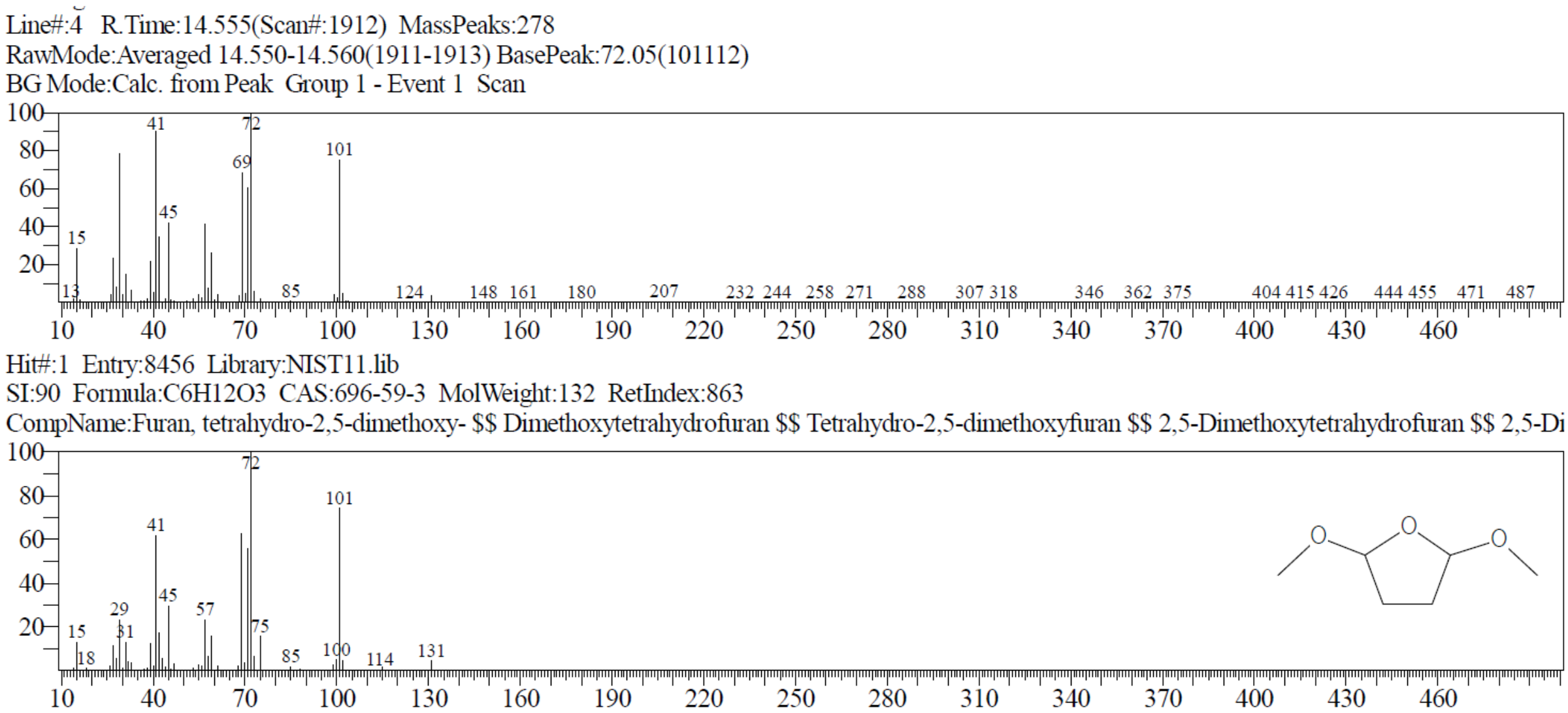

Figure S22: Mass spectrum of 2,5-dimethoxy-tetrahydrofuran and library reference spectrum

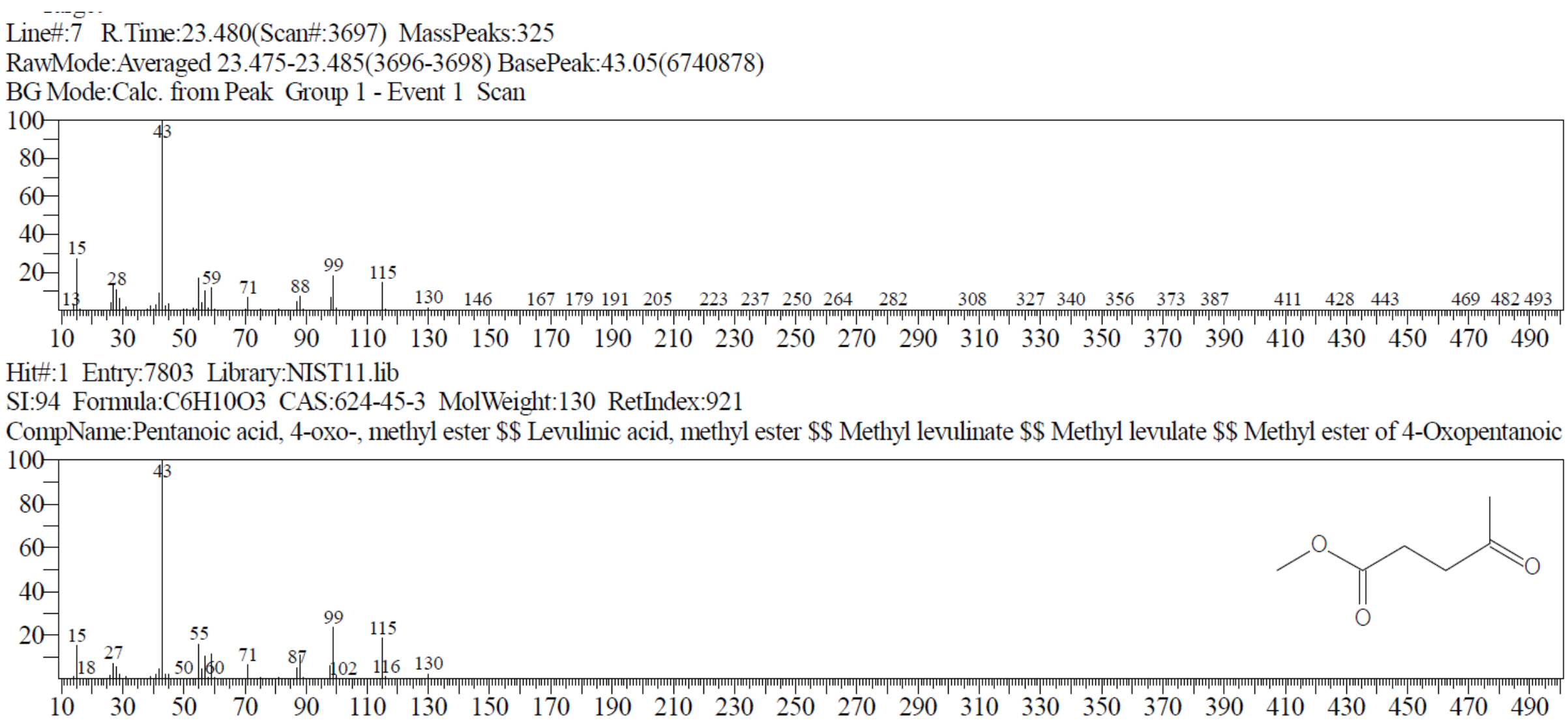

Figure S23: Mass spectrum of methyl levulinate and library reference spectrum

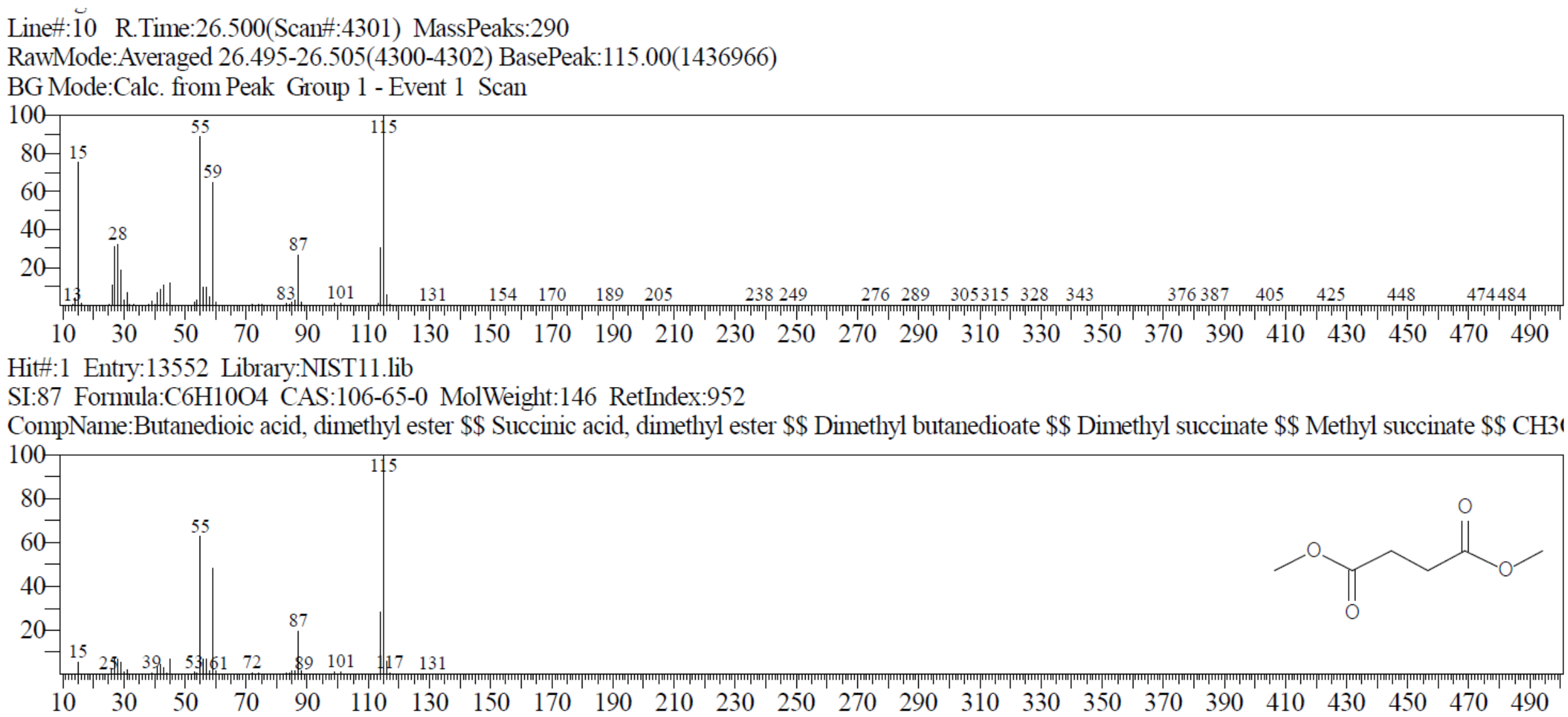

Figure S24: Mass spectrum of dimethyl succinate and library reference spectrum

GAS CHROMATOGRAPHY – FLAME IONIZATION DETECTION

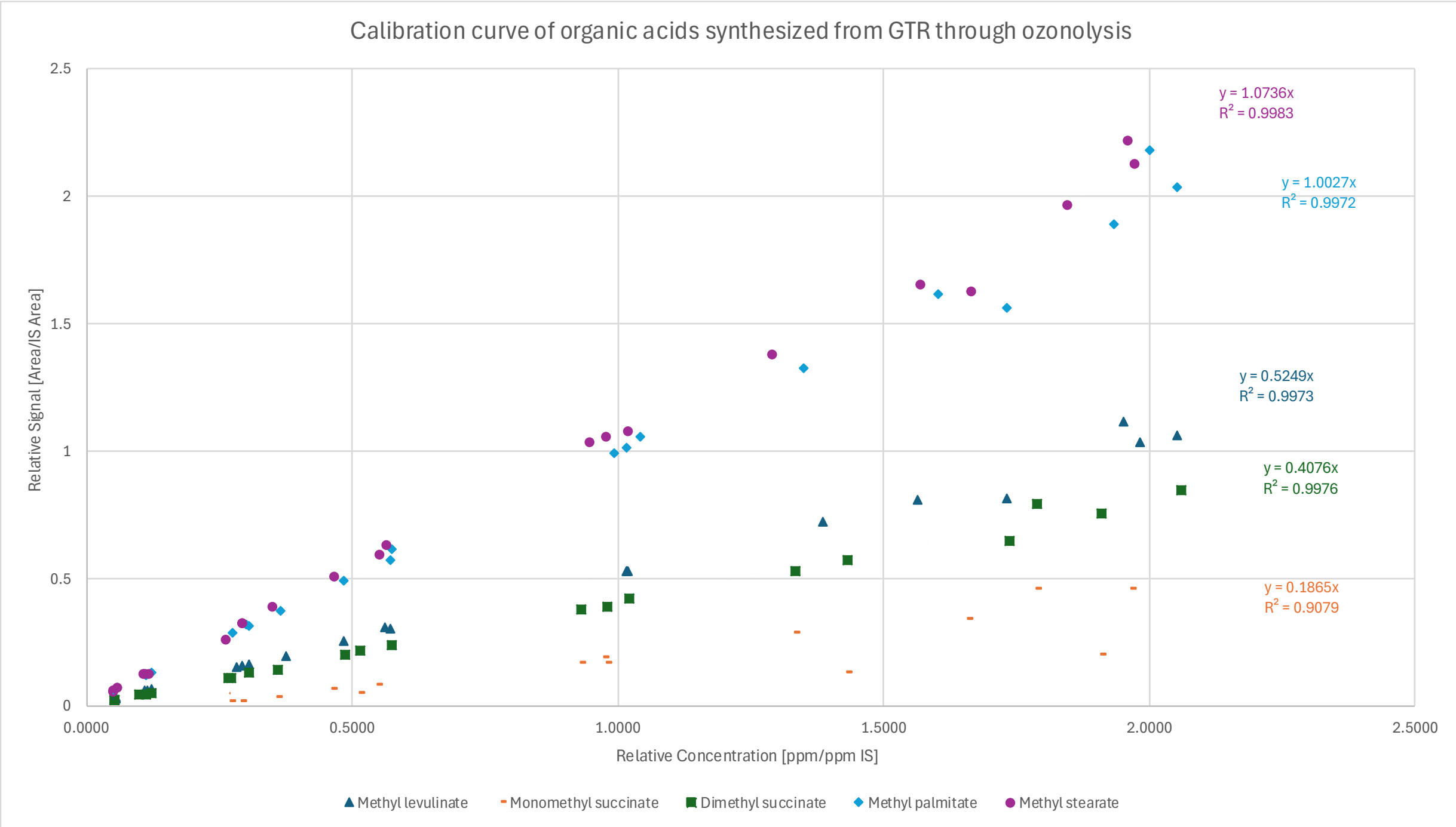

Figure S25: GC-FID 50-2000 ppm calibration curve of Methyl levulinate (blue, triangle); Monomethyl succinate (orange, dash), Dimethyl succinate (green, square), Methyl palmitate (light blue, diamond), Methyl stearate (purple, circle) relative concentration to 10000 ppm IS (methyl decanoate) to relative area

ENERGY DISPERSIVE X-RAY FLUORESCENCE

Measurement Condition					Collimator	3mm	Atmos.	He
Channel	kV	uA	Filter	Acq.	Analysis	Time	DT%	
Al-U	50	113-Auto	----	0 - 40	0.00-40.00	Live- 60	30	
C-Sc	15	1000-Auto	----	0 - 20	0.00- 4.40	Live- 100	30	
S-K	15	999-Auto	#2	0 - 20	2.10- 3.40	Live- 100	19	

Quantitative Result						
Analyte	Result		Std.Dev.	Calc.Proc	Line	Intensity
Cl	2.321	%	[0.024]	Quan-FP	ClKa	0.8349
S	1.301	%	[0.024]	Quan-FP	S Ka	0.2525
Zn	1.229	%	[0.003]	Quan-FP	ZnKa	292.5266
Fe	0.080	%	[0.001]	Quan-FP	FeKa	10.1529
Co	0.029	%	[0.000]	Quan-FP	CoKa	4.5143
Br	0.024	%	[0.000]	Quan-FP	BrKa	7.0655
Si	0.023	%	[0.002]	Quan-FP	SiKa	0.0315
Cu	0.018	%	[0.000]	Quan-FP	CuKa	3.7973
K	0.013	%	[0.001]	Quan-FP	K Ka	0.0139
Ca	0.007	%	[0.001]	Quan-FP	CaKa	0.0366
Mn	0.002	%	[0.000]	Quan-FP	MnKa	0.1452
CH2	94.955	%	[------]	Balance	--------	--------

Profile

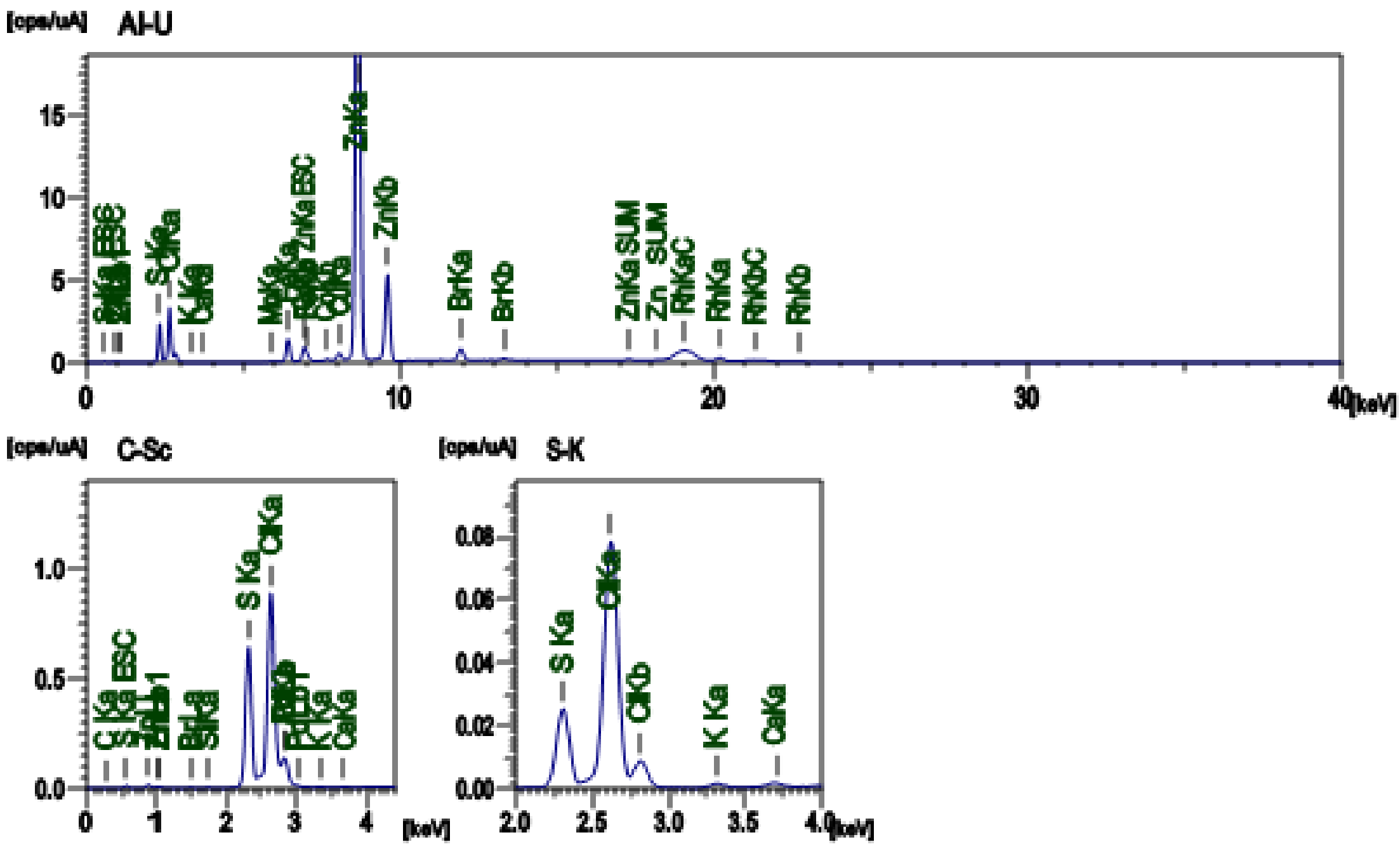

Figure S26: ED-XRF results of crude ozonolysis product indicating the presence of 1.2 wt% Zn
